# Mitochondrial PKM2 deacetylation by procyanidin B2-induced SIRT3 upregulation alleviates lung ischemia/reperfusion injury

**DOI:** 10.1038/s41419-022-05051-w

**Published:** 2022-07-11

**Authors:** Jing Zhao, Guorong Wang, Kaitao Han, Yang Wang, Lin Wang, Jinxia Gao, Sen Zhao, Gang Wang, Shengyang Chen, An Luo, Jianlin Wu, Guangzhi Wang

**Affiliations:** 1 Department of Pharmacy, Affiliated Zhongshan Hospital of Dalian University, Dalian, 116001 China; 2 Department of General Surgery, The Second Hospital of Dalian Medical University, Dalian, 116023 China; 3 Department of Clinical Laboratory, The Second Hospital of Dalian Medical University, Dalian, 116023 China; 4 Department of Anesthesiology, The Second Hospital of Dalian Medical University, Dalian, 116023 China; 5 Department of Thoracic Oncology, Affiliated Zhongshan Hospital of Dalian University, Dalian, 116001 China; 6 Department of Pharmacy, The Third Affiliated Hospital of Shenzhen University, Shenzhen, 518001 China; 7 Department of Radiology, Affiliated Zhongshan Hospital of Dalian University, Dalian, 116001 China

**Keywords:** Medical research, Respiratory tract diseases

## Abstract

Apoptosis is a critical event in the pathogenesis of lung ischemia/reperfusion (I/R) injury. Sirtuin 3 (SIRT3), an important deacetylase predominantly localized in mitochondria, regulates diverse physiological processes, including apoptosis. However, the detailed mechanisms by which SIRT3 regulates lung I/R injury remain unclear. Many polyphenols strongly regulate the sirtuin family. In this study, we found that a polyphenol compound, procyanidin B2 (PCB2), activated SIRT3 in mouse lungs. Due to this effect, PCB2 administration attenuated histological lesions, relieved pulmonary dysfunction, and improved the survival rate of the murine model of lung I/R injury. Additionally, this treatment inhibited hypoxia/reoxygenation (H/R)-induced A549 cell apoptosis and rescued Bcl-2 expression. Using *Sirt3*-knockout mice and specific *SIRT3* knockdown in vitro, we further found that SIRT3 strongly protects against lung I/R injury. *Sirt3* deficiency or enzymatic inactivation substantially aggravated lung I/R-induced pulmonary lesions, promoted apoptosis, and abolished PCB2-mediated protection. Mitochondrial pyruvate kinase M2 (PKM2) inhibits apoptosis by stabilizing Bcl-2. Here, we found that PKM2 accumulates and is hyperacetylated in mitochondria upon lung I/R injury. By screening the potential sites of PKM2 acetylation, we found that SIRT3 deacetylates the K433 residue of PKM2 in A549 cells. Transfection with a deacetylated mimic plasmid of PKM2 noticeably reduced apoptosis, while acetylated mimic transfection abolished the protective effect of PKM2. Furthermore, *PKM2* knockdown or inhibition in vivo significantly abrogated the antiapoptotic effects of SIRT3 upregulation. Collectively, this study provides the first evidence that the SIRT3/PKM2 pathway is a protective target for the suppression of apoptosis in lung I/R injury. Moreover, this study identifies K433 deacetylation of PKM2 as a novel modification that regulates its anti-apoptotic activity. In addition, PCB2-mediated modulation of the SIRT3/PKM2 pathway may significantly protect against lung I/R injury, suggesting a novel prophylactic strategy for lung I/R injury.

## Introduction

Lung ischemia/reperfusion (I/R) injury is a life-threatening disorder that occurs in patients subjected to lung transplantation, cardiopulmonary bypass, cardiac surgery, and pulmonary embolism [1–3]. Pathologically, lung I/R injury is characterized by nonspecific alveolar epithelial damage, lung edema, pulmonary hypertension and hypoxemia, which contribute to the high mortality rate [2, 4, 5]. Programmed pulmonary epithelial cell death, such as apoptosis, is a critical event during the pathophysiological process of lung I/R injury [6, 7]. A previous study suggested that when rat donor lungs are preserved for up to 12 h, apoptosis is the major mode of cell death after transplantation; in human lung transplantation, up to 34% of pulmonary cells undergo apoptotic cell death after reperfusion [6–8]. Furthermore, administration of a caspase inhibitor or small interfering RNA (siRNA) targeting *Fas* is known to protect the lung from I/R injury [9]. Therefore, antiapoptotic strategies may be an attractive approach to improve the outcomes of patients with lung I/R injury.

Pyruvate kinase is a rate-limiting enzyme that acts at the final step of glycolysis and catalyzes the conversion of phosphoenolpyruvate to pyruvate [10]. In mammals, the four isoforms of pyruvate kinase are encoded by two distinct genes, *PKLR* and *PKM*. *PKLR* encodes pyruvate kinase L and R, which are expressed in the liver and erythrocytes, respectively. Alternative splicing of the *PKM* pre-mRNA leads to the generation of pyruvate kinase M1 and pyruvate kinase M2 (PKM2), both of which are widely expressed in various types of cells and tissues [10, 11]. Among these isoforms, PKM2 has been the most extensively studied in many key biological processes, including cell proliferation, metabolism, inflammation and especially apoptosis [12–14]. An increasing number of reports have confirmed that PKM2 functions as an antiapoptotic factor. Depletion of the *PKM2* mRNA with an siRNA resulted in decreased viability and increased apoptosis in multiple cancer cell lines. In non-small cell lung cancer, silencing of *PKM2* enhanced ionizing radiation-induced apoptosis in vitro and in vivo [15]. Recently, a more detailed study revealed that PKM2 translocates to the mitochondria and inhibits Bcl-2 degradation, resulting in apoptotic resistance after hydrogen peroxide insult [16]. Given the pivotal role of apoptosis in the pathogenesis of lung I/R injury, we hypothesize that PKM2 may provide effective protection against this condition by regulating apoptosis.

Acetylation of lysine 433 is a classic posttranslational modification critical for PKM2 destabilization and intracellular relocalization [17]. Although PKM2 inhibits Bcl-2 degradation and regulates apoptosis in the mitochondria, researchers have not clearly determined whether intramitochondrial acetylation of PKM2 is involved in the regulation of apoptosis and protection against lung I/R injury. Previous studies have suggested that sirtuin 3 (SIRT3), a nicotinamide adenine dinucleotide (NAD^+^)-dependent deacetylase, is the only sirtuin predominantly localized in mitochondria that exhibits strong deacetylase activity [18, 19]. More than 65% of the total mitochondrial proteins are acetylated, and SIRT3 is the most important deacetylase catalyzing their deacetylation [18]. Importantly, SIRT3 modulates various important nonhistone substrates, including acetyl coenzyme A synthetase, manganese superoxide dismutase 2 and isocitrate dehydrogenase [20–22]. Because of its crucial targets, SIRT3 regulates diverse physiological processes, ranging from lipid metabolism, redox homeostasis, and inflammatory response to apoptosis [19, 20, 22, 23]. SIRT3 upregulation by melatonin significantly inhibits I/R-induced cardiac dysfunction and apoptosis by activating superoxide dismutase 2 [24]. In our recent study, we found that SIRT3-mediated deacetylation of peroxiredoxin 3 substantially increased its activity and reduced intestinal I/R-induced epithelial apoptosis [23]. Although a previous study suggested that SIRT3 signaling may be involved in rats with lung I/R injury and type II diabetes [25], the detailed mechanisms, as well as whether PKM2 functions as an antiapoptotic substrate of SIRT3, require further investigation.

Traditionally, calorie restriction and endurance exercise are considered the only two available methods to increase mitochondrial SIRT3 expression [19]. However, those two approaches are not feasible for every patient prepared for a cardiopulmonary operation. In our preliminary experiments, procyanidin B2 (PCB2) showed a stronger ability to upregulate the *SIRT3* mRNA in the mouse lung than other common polyphenols (Supplementary Fig. 1a, b). In addition, several studies have suggested that PCB2 administration protects against acute lung injury in rats or human type II alveolar epithelial cells [26, 27]. Therefore, we hypothesized that PCB2 may activate SIRT3 in the lung, and this effect might be beneficial for preventing I/R-induced lung injury.

In this study, we first report that the SIRT3/PKM2 pathway is a protective target for the suppression of apoptosis in lung I/R injury. Moreover, the K433 residue might be the dominant acetylation site on PKM2 that is involved in SIRT3-mediated antiapoptotic activity. This work aims to further delineate the mechanisms of lung I/R injury and to provide a novel prophylactic target for patients with lung I/R injury.

## Materials and methods

### Experimental lung I/R model and drug treatment

Adult male C57BL/6 mice, weighing 20 ± 2 g at 8 weeks of age, were obtained from the Laboratory Animal Center of Dalian Medical University (Dalian, China). *Sirt3*^-/-^ mice were purchased from Cyagen Bioscience Incorporated Company (Guangzhou, China). All mice used in this study were randomly assigned to groups. Anesthesia and analgesia management and an in vivo hilar clamp model of lung I/R were established as previously described [28, 29]. Briefly, mice were initially anesthetized with an intraperitoneal injection of sodium pentobarbital (50 mg/kg) and placed on a heating pad to maintain their body temperature. Buprenorphine (0.1 mg/kg) was injected subcutaneously prior to the skin incision to minimize pain, distress or discomfort. After we ensured an appropriate depth of anesthesia, the mice were intubated *via* tracheotomy and connected to a rodent ventilator (RWD Life Science, Shenzhen, China) with tidal volumes of 10 μl/g and a respiratory rate set to 120 breaths per minute. After thoracotomy, the left hilum of the I/R group was occluded with an atraumatic microclamp for 1 h of unilateral ischemia and then released for 2 h of reperfusion. In animals on single-lung ventilation, the tidal volume was reduced to 6 μl/g, and the respiratory rate was increased to 150 breaths per minute. Sham animals received the same anesthesia and surgery but without hilar occlusion, as described in a previous report [29].

For drug administration, PCB2 (30 or 15 mg/kg, purity: 99.40%, MedChemExpress, Monmouth Junction, NJ, USA), compound 3 K (10 mg/kg, purity: 99.66%, Selleck, Houston, TX, USA), curcumin (25 mg/kg, purity: 99.83%, Selleck), (-)-epigallocatechin gallate (EGCG, 85 mg/kg, purity: 99.68%, Selleck), protocatechuic acid (PCA, 30 mg/kg, purity: 99%, J&K Scientific Ltd., Beijing, China), quercetin (50 mg/kg, purity: 98.39%, Selleck), resveratrol (10 mg/kg, purity ≥ 99%, Sigma-Aldrich, St. Louis, MO, USA) or vehicle was injected intraperitoneally once a day for three consecutive days before the operation. These dosages were consistent with those used in previous studies [14, 30–35]. The mice were sacrificed at the end of the experiments, and blood and lung samples were collected for further analyses.

All experiments were performed in accordance with the National Institutes of Health Guide for the Care and Use of Laboratory Animals and were approved by the Institutional Ethics Committee of Affiliated Zhongshan Hospital of Dalian University.

### Histological analysis and immunohistochemical staining

For histological analyses, tissues were fixed and embedded in paraffin. Consecutive sections with a thickness of 4 *μ*m were stained with hematoxylin and eosin (H&E). Histopathological scores were assigned to the lung based on the histological scoring systems described by Mikawa [36].

Immunohistochemistry (IHC) for SIRT3 was performed on paraffin sections with a thickness of 4 μm. Sections were incubated with a SIRT3 antibody (#2627, Cell Signaling Technology, MA, USA) overnight and visualized with diaminobenzene (Sigma-Aldrich). The number of positive cells was quantified with ImageJ software (National Institutes of Health, Bethesda, MD, USA).

### Apoptosis assay

Terminal-deoxynucleotidyl transferase mediated nick end labeling (TUNEL) staining was conducted with an in situ apoptosis detection kit (Roche, Branchburg, NJ, USA) according to the manufacturer’s protocol. A GreenNuc Living Cell Caspase-3 Activity Detection Kit (Beyotime Institute of Biotechnology, Shanghai, China) was used to detect caspase-3 activity in living cells according to the manufacturer’s instructions. Caspase-3 activity in lung tissues was examined using a caspase-3 activity assay kit (Beyotime Institute of Biotechnology) according to the manufacturer’s protocol. Briefly, caspase-3 catalyzes the transformation of Ac-DEVD-pNA (acetyl-Asp-Glu-Val-Asp p-nitroanilide) to yellow pNA (p-nitroanilide), which can be measured at 405 nm. A fluorescence-activated cell sorting (FACS) Apoptosis Detection Kit (Beyotime Institute of Biotechnology) containing fluorescein isothiocyanate (FITC)-Annexin V and propidium iodide (PI) was employed to measure the cell apoptosis rate. Fluorescence was measured using an Agilent NovoCyte Advanteon instrument (Agilent, Santa Clara, CA, USA).

### Survival analysis

After a 2-h reperfusion, the mice were ventilated and observed for an additional 1 h to estimate the survival rate. During this period, the contralateral hilum (right hilum) was permanently ligated to ensure that the animal’s survival and gas exchange depended solely upon the reperfused lung [37]. The definition of death was a combination of (i) cardiac arrest or cessation of regular cardiac activity, (ii) the collapse of the left atrium, and (iii) brief clonus activity [28]. A researcher blinded to the group assignment recorded the death events every five minutes for the survival analysis.

### Cell culture and hypoxia/reoxygenation (H/R) treatment

The A549 cell line, which is derived from a human alveolar cell carcinoma, has many properties of human alveolar epithelial cells and was utilized because it is the most widely used in vitro model of type II pulmonary alveolar epithelial cells [38]. The cells were obtained from the American Type Culture Collection (ATCC^®^ CCL-185^TM^) and were maintained in Dulbecco’s modified Eagle’s medium supplemented with 10% fetal bovine serum (Gibco, Carlsbad, CA, USA) and 1% glutamine in a humidified atmosphere with 5% CO_2_ at 37 °C. Before the experiments, the cells were authenticated by short tandem repeat profiling and tested for mycoplasma contamination. For H/R conditions, Cells were exposed to a microaerophilic system (Thermo Fisher Scientific 8000, Marietta, GA) with 5% CO_2_ and 1% O_2_ balanced with 94% N_2_ at 37 °C for 6 h followed by reoxygenation in 95% air and 5% CO_2_ for 2 h to establish H/R conditions, as described in previous reports with a few modifications [28]. For 3-(1H-1,2,3-triazol-4-yl) pyridine (3-TYP, Selleck), nicotinamide (NAM, Sigma-Aldrich) or trichostatin A (TSA, Sigma-Aldrich) treatment, the cells were incubated with 50 μM 3-TYP, 10 mM NAM or 0.5 μM TSA for the indicated times and then subjected to H/R, as needed [23].

### Lung wet to dry (W/D) ratios

The ratio of lung W/D weight was calculated to quantify the degree of pulmonary edema. The left upper lobe of the lung was cleansed and weighed to obtain the wet weight. The lung was then placed in an oven at 80 °C for 12 h to obtain the dry weight and calculate the W/D weight ratio.

### Analysis of the protein concentration in bronchoalveolar lavage fluid (BALF)

The lungs were lavaged using normal saline *via* tracheal cannulation to obtain the BALF. The protein concentrations in BALF were determined using a Bradford Protein Quantification Kit (Beyotime Institute of Biotechnology) according to the manufacturer’s instructions.

### Lung myeloperoxidase (MPO) activity assay

Lung tissues were homogenized by adding a cold isotonic sodium chloride solution and centrifuged at 3 000 × g for 15 minutes to remove the debris. MPO activity was determined using a commercial biochemical kit (Nanjing Jiancheng Bioengineering Institute, Nanjing, China) according to the manufacturer’s instructions.

### Arterial blood gas analysis

Arterial blood was collected with a heparinized syringe from the anesthetized mice at the end of the experiments. An automated blood gas analyzer (Shanghai Shengke Instrument Co., Ltd., Shanghai, China) was used to measure partial arterial oxygen pressure (PaO_2_), partial arterial carbon dioxide pressure (PaCO_2_) and PaO_2_/fraction of inspired oxygen (FiO_2_).

### Cell viability assay

Cell viability was quantitatively analyzed using the 3-(4,5-dimethyl-2-thiazolyl)-2,5-diphenyl-2H-tetrazolium bromide (MTT) assay (Sigma-Aldrich). Briefly, the experiments were conducted in 96-well plates, and the absorbance of each individual well was determined at 570 nm.

### Cell transfection

The PKM2 and SIRT3 expression plasmids, as well as the PKM2 mutant plasmid, were synthesized by GenePharma (Shanghai, China). The SIRT3-H248Y plasmid was obtained from Addgene (Watertown, MA, USA). Briefly, A549 cells were transfected with 2 μg of the expression plasmid or the negative control vector using Lipofectamine 3000 (Invitrogen, Carlsbad, CA, USA). For siRNA transfection, cells were transfected with specific or control siRNAs (GenePharma) using Lipofectamine 3000 for 48 h. The sequences of the siRNAs were as follows: SIRT3, sense 5’-GAA ACU ACA AGC CCA ACG UTT-3’ and antisense 5’-ACG UUG GGC UUG UAG UUU CTT-3’; PKM2 sense 5’- CAU CUA CCA CUU GCA AUU ATT-3’ and antisense 5’- UAA UUG CAA GUG GUA GAU GTT-3’; and negative control, sense 5’-UUC UCC GAA CGU GUC ACG UTT-3’ and antisense 5’ ACG UGA CAC GUU CGG AGA ATT-3’.

### Western blot analysis

Total proteins or mitochondrial proteins were extracted using commercial kits (Beyotime Institute of Biotechnology). Western blotting was performed with antibodies against SIRT3 (Cell Signaling Technology), Bcl-2 (#AB112, Beyotime Institute of Biotechnology), PKM2 (#ab137791, Abcam Cambridge, UK), β-actin (#20536-1-AP, Proteintech, Wuhan, China), Tubulin (#T6199, Sigma-Aldrich) and VDAC (#10866-1-AP, Proteintech Group). Protein quantification was performed using ImageJ software.

### Immunoprecipitation analysis

Total proteins or mitochondrial proteins were incubated with equal amounts of the anti-PKM2 antibody at 4 °C overnight after removing debris by centrifugation at 4 °C. Next, the immunocomplex was captured by adding Protein A + G magnetic beads (Bimake, Houston, TX, USA) according to the manufacturer’s protocols. The precipitate was washed five times with washing buffer. The samples were resuspended in 1× loading buffer and boiled for 10 minutes to dissociate the immunocomplex from the beads. Finally, the supernatant was collected by magnetic separation and subjected to Western blotting with anti-SIRT3 or anti-acetyl lysine antibodies (#ab21623, Abcam, Ltd. and then incubated with an anti-IgG heavy chain-specific secondary antibody (#A25222, Abbkine, Wuhan, China).

### Immunofluorescence staining

A549 cells were treated with 200 nM MitoTracker CMXRos (Invitrogen) and incubated with anti-PKM2 and the secondary antibody as described in our previous studies [23, 39]. For dual immunofluorescence staining, cells were incubated with the primary antibody at 4 °C overnight followed by an incubation with the secondary antibody (Proteintech) at 37 °C for 1 h. DAPI was used for nuclear staining. Immunofluorescence images were collected under an inverted fluorescence microscope. Colocalization data were quantified using ImageJ software.

### Quantitative real-time polymerase chain reaction

Total RNA was extracted using RNAiso Plus (TaKaRa, Kusatsu, Japan), and cDNA synthesis and RNA amplification were performed with a PrimeScript RT reagent kit and SYBR Premix Ex Taq II (TaKaRa), respectively. Expression levels in each sample normalized to that of β-actin were determined by calculating ΔΔCt. Sequences of the primers used in the present study are listed in Supplementary Table 1. Experimental validation data for all primers are available from PrimerBank (https://pga.mgh.harvard.edu/primerbank/).

### Statistical analysis

All experiments were repeated independently at least three times. The results in this study are presented as the means ± standard deviations. When the data displayed a normal distribution or lognormal distribution after the Shapiro-Wilk test, statistical comparisons between multiple groups were analyzed with one-way analysis of variance followed by Tukey’s test for data with homogenous variances or followed by Dunnett’s T3 test for data with heterogeneity of variance. The two-tailed Student’s *t*-test was used to compare two groups with normally distributed data and homogenous variances; if variances were heterogeneous, Welch’s *t*-test was used. Ranked data were analyzed using the Kruskal–Wallis rank test. The product limit (Kaplan–Meier) estimate of the cumulative survival was assessed with the log-rank test to evaluate significant differences in survival. All statistical analyses were performed using GraphPad Prism 8.0 (GraphPad Prism Software, La Jolla, CA, USA). *P* values less than 0.05 were considered statistically significant. The sample size was estimated from our preliminary experiments or from previous reports [23, 28].

## Results

### PCB2 protects mice from lung I/R injury

**Fig. 1:**
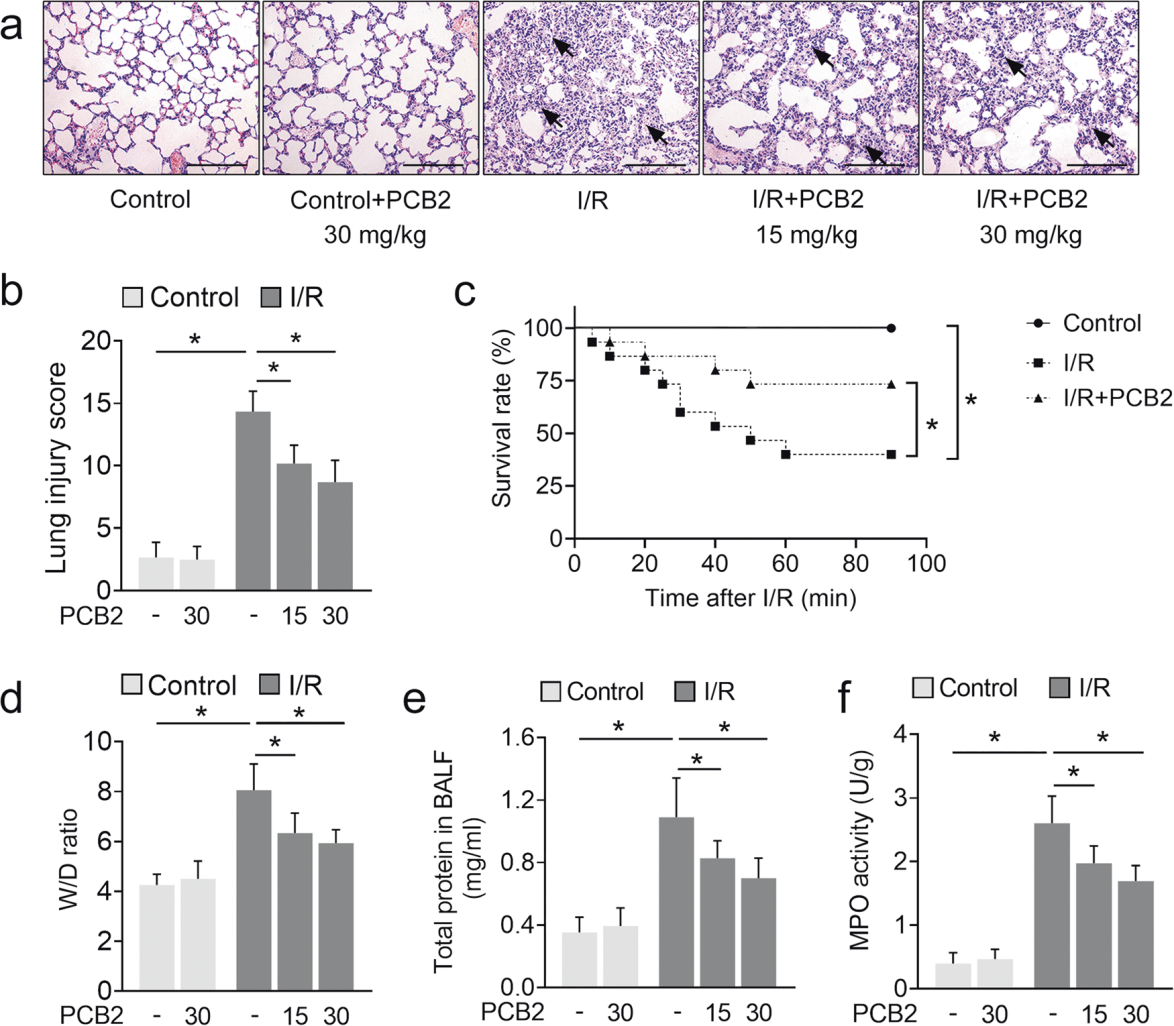
PCB2 protects against lung I/R injury in mice. **a**–**f** C57BL/6 mice were subjected to 1 h of ischemia followed by 2 h of reperfusion to establish lung I/R injury; mice from the control group underwent sham surgical procedures. The indicated dosages of PCB2 were injected intraperitoneally for three consecutive days before the operation. **a** Representative images of the H&E-stained lung tissue. Scale bar = 200 μm. Black arrows indicate representative pathological damage. **b** Histopathological scores (Mikawa’s score) of the lung, *n* = 6. The dosage of PCB2 was 15 or 30 mg/kg, as indicated. **c** The survival rate of the mice, *n* = 15. The dosage of PCB2 was 30 mg/kg. **d**–**f** The W/D ratio, total protein concentration in BALF and MPO activity in the lung, *n* = 6. The dosage of PCB2 was 15 or 30 mg/kg, as indicated. *p < 0.05. Error bars depict the standard deviations.

In our preliminary experiments, PCB2 showed a potent ability to activate SIRT3 in the mouse lung (Supplementary Fig. 1a). We established a stable and reproducible mouse model and pretreated the medication group with PCB2 at a graded dose of 15 or 30 mg/kg for three consecutive days before I/R injury to determine whether PCB2 administration was a beneficial treatment for lung I/R injury. Pulmonary histopathological changes, arterial blood gases and the survival rate were examined. As shown in Fig. 1a, H&E staining revealed that lung I/R injury induced pulmonary capillary congestion, interstitial edema, inflammatory cell infiltration and alveolar wall thickening. Correspondingly, the quantitative score of histological injury was substantially increased (Fig. 1b). In contrast, in the medication groups, PCB2 attenuated lung histopathological damage and resulted in a significant decrease in quantitative scores. In addition, the arterial blood gas analysis of the I/R-treated mice showed significant changes, with a decrease in PaO_2_, an increase in PaCO_2_ and a decrease in PaO_2_/FiO_2_ (Table 1, upper panel). However, PCB2 effectively mitigated these changes to improve the arterial oxygenation index. In the I/R + PCB2 groups, PaO_2_ and PaO_2_/FiO_2_ were significantly increased, along with a decrease in PaCO_2_ (Table 1, upper panel). Moreover, the survival rate of animals in the lung I/R injury group was significantly lower than that in the control group but was significantly increased by PCB2 treatment (Fig. 1c).

**Table 1:** Comparison of PaO_2_, PaCO_2_ and PaO_2_/FiO_2_ in each group of mice.

Group	Dosage (mg/kg)	PaO_2_ (mmHg)	PaCO_2_ (mmHg)	PaO_2_/FiO_2_ (mmHg)
Control	−	93.13 ± 5.49	40.03 ± 3.11	443.49 ± 26.13
Control+PCB2	30	92.79 ± 5.02	41.18 ± 2.78	441.83 ± 23.89
I/R	−	55.12 ± 5.53*	61.62 ± 5.91*	262.45 ± 26.35*
I/R + PCB2	15	69.69 ± 4.49^#^	56.60 ± 4.85	331.87 ± 21.39^#^
I/R + PCB2	30	76.13 ± 6.45^#^	50.74 ± 4.63^#^	362.52 ± 30.71^#^
Control (*Sirt3* ^+/+^)	−	94.03 ± 1.63	40.58 ± 2.60	447.79 ± 7.76
I/R (*Sirt3* ^+/+^)	−	57.13 ± 3.36^@^	61.40 ± 5.13^@^	272.07 ± 15.98^@^
I/R + PCB2 (*Sirt3* ^+/+^)	−	76.78 ± 4.78^&^	49.28 ± 4.50^&^	365.63 ± 22.77^&^
I/R (*Sirt3* ^-/-^)	−	50.32 ± 4.85	69.87 ± 5.70	239.61 ± 23.11
I/R + PCB2 (*Sirt3* ^-/-^)	−	52.77 ± 3.53	65.97 ± 6.13	251.27 ± 16.81

*p < 0.05 *versus* Control group, ^#^*p* < 0.05 *versus* I/R group, ^@^*p* < 0.05 *versus* Control (*Sirt3* ^+/+^) group, ^&^*p* < 0.05 *versus* I/R (*Sirt3* ^+/+^) group. *n* = 6.

The lung W/D ratio and BALF protein concentration are two commonly used indicators of pulmonary vascular permeability and are important characteristics of acute lung injury. The W/D ratio and BALF protein concentration of the lung I/R-challenged group were substantially increased compared with those of the control group, and these levels were gradually decreased by graded PCB2 treatment (Fig. 1d, e). We also detected the activity of MPO, an indicator of neutrophil infiltration, in the lung. Compared with the control group, MPO activity in the I/R-stimulated lungs was substantially increased, but was inhibited by PCB2 treatment. (Fig. 1f). Collectively, these results indicated that PCB2 effectively protects against lung I/R injury.

### PCB2 alleviates pulmonary epithelial apoptosis induced by lung I/R injury

**Fig. 2:**
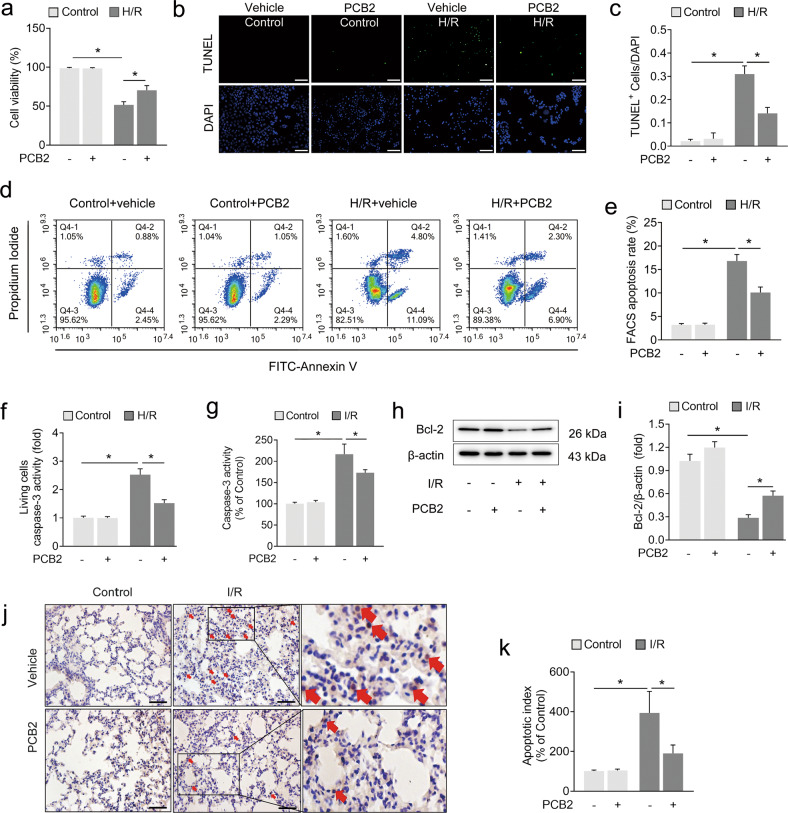
PCB2 alleviates I/R- or H/R-induced pulmonary cell apoptosis. **a**–**f** A549 cells were treated with 20 μM PCB2 for 24 h and then subjected to 6 h of hypoxia followed by 2 h of reoxygenation to achieve H/R injury. **a** Cell viability, *n* = 6. **b** Representative images of TUNEL staining in A549 cells; the nuclei were stained with DAPI. Scale bar = 200 μm. **c** TUNEL scores. The relative number of apoptotic cells is presented as TUNEL-positive cells/DAPI (*n* = 6). **d** Representative FACS dot plots of annexin V and propidium iodide dual labeling. **e** FACS analysis of the apoptotic rate in A549 cells. The sum of the annexin V-positive population and propidium iodide-positive population is shown. *n* = 3. **f** Caspase-3 activity in living A549 cells. *n* = 6. **g**–**k** C57BL/6 mice were subjected to 1 h of ischemia followed by 2 h of reperfusion to establish lung I/R injury; mice from the control group underwent sham surgical procedures. PCB2 (30 mg/kg) was injected intraperitoneally for three consecutive days before the operation. **g** Caspase-3 activity in the lung, *n* = 6. **h**, **i** Expression of the Bcl-2 protein in the lung, *n* = 3. **j** Representative images of TUNEL staining in the lung. Scale bar = 100 μm. **k** Apoptotic index of the lung, *n* = 3. *p < 0.05. Error bars depict the standard deviations.

We established a commonly used H/R model that simulates lung ischemia and blood restoration to determine the molecular and cytopathological changes induced by I/R using previously reported methods with a few modifications [28]. As shown in Fig. 2a, H/R treatment led to a decrease in cell viability compared with the control group, and PCB2 significantly improved cell viability. Pulmonary epithelial cell apoptosis is the major mode of cell death involved in the pathogenesis of lung I/R injury [6, 7]. We determined the effect of PCB2 on apoptosis by performing TUNEL staining and FACS analysis of apoptotic cells. As shown in Fig. 2b–e, the proportion of TUNEL-positive nuclei and the apoptosis rate detected using FACS were significantly increased in the H/R group compared to those of the control group. Moreover, apoptosis was further confirmed by performing a living cell caspase-3 activity assay (Fig. 2f). PCB2 reduced the proportion of TUNEL-positive cells and apoptosis rate detected using FACS and inhibited caspase-3 activation (Fig. 2b–f). Furthermore, TUNEL staining, caspase-3 activity assays and Bcl-2 western blot analysis were performed to determine whether the apoptosis observed in vitro occurred in vivo. As shown in Fig. 2g–k, I/R injury led to significant increases in caspase-3 activation, decreased Bcl-2 expression and increased numbers of TUNEL-positive cells in the lung. Similarly, these effects were considerably alleviated by the PCB2 pretreatment. Thus, lung I/R injury causes apoptotic cell death, which is clearly attenuated by PCB2 administration.

### PCB2 relieves I/R-induced SIRT3 suppression in the lung

**Fig. 3:**
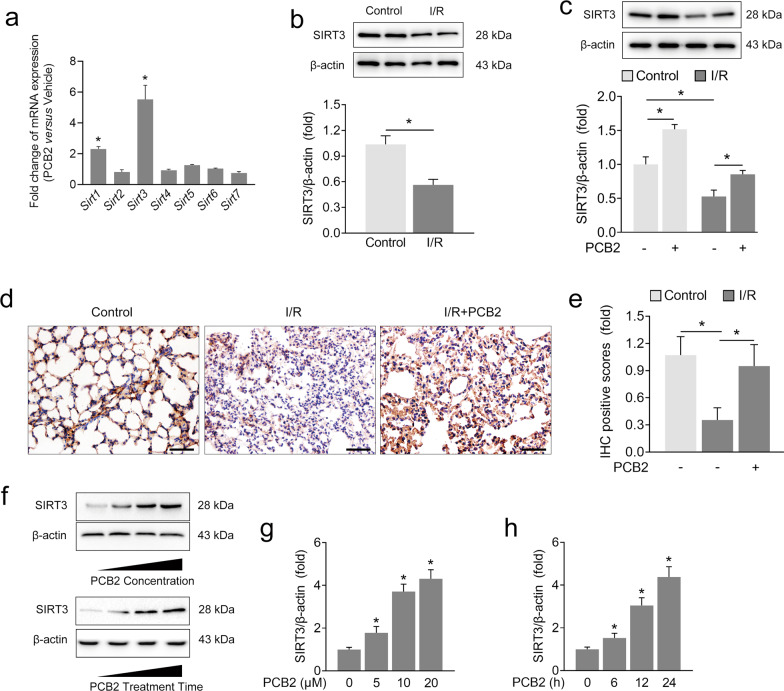
PCB2 attenuates I/R-induced SIRT3 suppression in the lung. **a** C57BL/6 mice were intraperitoneally injected with 30 mg/kg PCB2 for three consecutive days. Fold changes in *Sirt1-7* mRNA expression in the lung, *n* = 3. *p < 0.05. **b**–**e** C57BL/6 mice were subjected to 1 h of ischemia followed by 2 h of reperfusion to establish lung I/R injury; mice from the control group underwent sham surgical procedures. PCB2 (30 mg/kg) was injected intraperitoneally for three consecutive days before the operation. **b**, **c** SIRT3 protein expression in the lung, *n* = 3. *p < 0.05. **d**, **e** IHC staining for SIRT3. Scale bar = 100 μm. *n* = 3. *p < 0.05. **f** [upper panel] **g** A549 cells were treated with the indicated concentrations of PCB2 for 24 h. SIRT3 protein expression in A549 cells, *n* = 3. **f** [lower panel] and **h** A549 cells were treated with 20 μM PCB2 for the indicated times. SIRT3 protein level in A549 cells, *n* = 3. *p < 0.05 compared with the first group. Error bars depict the standard deviations.

Sirtuins are a family of NAD^+^-dependent histone deacetylases implicated in various cellular processes. Our previous studies verified the antiapoptotic properties of different sirtuins in various organs after I/R injury [23, 30, 40]. Given the antiapoptotic effects of PCB2 on lung I/R injury, we investigated whether sirtuin activation was associated with PCB2 administration in mice. As shown in Figs. 3a, 2 sirtuin mRNAs were significantly upregulated (*Sirt1* and *Sirt3*) in the lung after PCB2 treatment. Other sirtuin mRNAs, including *Sirt2, Sirt4, Sirt5, Sirt6 and Sirt7*, showed nonsignificant changes. Among the sirtuins showing significant changes in mRNA expression, *Sirt3* expression was increased most substantially by 5.53-fold. Next, we investigated SIRT3 upregulation following PCB2 treatment in mice with lung I/R injury. As shown in Fig. 3b, c, a decrease in SIRT3 protein expression was observed in the lung I/R injury group compared with the control group. However, PCB2 prevented the I/R-induced decrease in SIRT3 expression (Fig. 3c). As expected, IHC staining for SIRT3 showed a similar trend (Fig. 3d, e). Additionally, PCB2 administration exerted similar effects on the in vitro model of H/R injury as in vivo (Supplementary Fig. 2). We evaluated SIRT3 protein expression in A549 cells administered PCB2 at concentrations of 0, 5, 10, or 20 μM for 24 h to further confirm the relationship between SIRT3 expression and PCB2 administration (Fig. 3f, g). Interestingly, a progressive increase in SIRT3 expression was detected, revealing that PCB2 may induce SIRT3 expression in a dose-dependent manner. Furthermore, when A549 cells were treated with 20 μM PCB2 for different durations, a gradual upregulation of SIRT3 expression was detected, as expected (Fig. 3f, h). These findings suggested that SIRT3 may be associated with lung I/R injury and that PCB2 attenuates the decrease in pulmonary SIRT3 expression induced by lung I/R injury.

### SIRT3 plays an indispensable role in PCB2-mediated protection against lung I/R injury

**Fig. 4:**
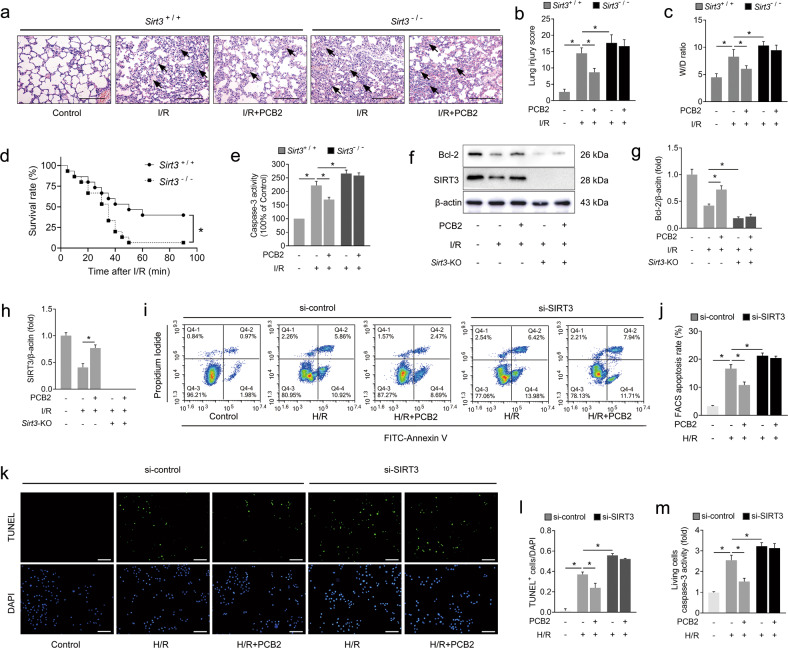
SIRT3 deficiency abolishes PCB2-mediated protection against lung I/R injury in vivo and in vitro. **a**–**h** I/R mice were subjected to 1 h of ischemia followed by 2 h of reperfusion to induce lung I/R injury. Mice from the control group underwent sham surgical procedures. PCB2 (30 mg/kg) was injected intraperitoneally for three consecutive days before the operation. *Sirt3*^+/+^ indicates *Sirt3* wild-type mice; *Sirt3*^-/-^ indicates *Sirt3*-knockout mice. **a** Representative images of H&E-stained lung tissues. Scale bar = 200 μm. Black arrows indicate representative pathological damage. **b** Histopathological scores (Mikawa’s score) of the lung, *n* = 6. **c** The W/D ratio of the lung, *n* = 6. **d** The survival rate of the I/R mice, *n* = 15. **e** Caspase-3 activity in the lung, *n* = 6. **f**–**h** Bcl-2 and SIRT3 protein levels in the lung, *n* = 3. *Sirt3*-KO indicates *Sirt3-*knockout mice. **i**–**m** A549 cells were transfected with an siRNA targeting *SIRT*3 (si-SIRT3) or the negative control (si-control), as indicated, before the H/R insult. Moreover, the cells from the PCB2 groups were treated with 20 μM PCB2 for 24 h. The cells from the H/R group were subjected to 6 h of hypoxia followed by 2 h of reoxygenation. **i** Representative FACS dot plots of annexin V and propidium iodide dual labeling. **j** FACS analysis of the apoptotic rate in A549 cells. The sum of the annexin V-positive population and propidium iodide-positive population is shown. *n* = 3. **k** Representative images of TUNEL staining in A549 cells; the nuclei were stained with DAPI. Scale bar = 200 μm. **l** TUNEL scores. The relative number of apoptotic cells is presented as TUNEL-positive cells/DAPI (*n* = 6). **m** Caspase-3 activity in living A549 cells. *n* = 6. *p < 0.05. Error bars depict the standard deviations.

We used *Sirt3*^*-/-*^ mice to establish a lung I/R model in vivo and to determine whether SIRT3 plays an important role in PCB2-mediated protection. Compared with the wild-type mice, *Sirt3*-deficient showed more serious histological injury, worse pulmonary function and a higher W/D ratio in the lung after I/R injury (Fig. 4a–c and Table 1, lower panel). In the groups with wild-type SIRT3 expression, PCB2 exerted protective effects similar to those described above (Fig. 4a–c and Table 1, lower panel). Interestingly, these protective effects were strongly abrogated by *Sirt3* knockout (Fig. 4a–c and Table 1, lower panel). Furthermore, *Sirt3*^*-/-*^ mice exhibited a lower survival rate than wild-type mice after lung I/R injury (Fig. 4d), and, as expected, PCB2 did not exert the same protective effects on *Sirt3*^*-/-*^ mice (Supplementary Fig. 3).

We subsequently detected caspase-3 activity and Bcl-2 expression in the lung to further explore the role of SIRT3 in PCB2-regulated apoptosis. As shown in Fig. 4e, the trend of caspase-3 activity was similar to the histological changes or the W/D ratio. Furthermore, Bcl-2 expression was obviously decreased upon I/R injury, and this effect was further enhanced by *Sirt3* knockout (Fig. 4f–h). In the *Sirt3* wild-type groups, PCB2 ameliorated the I/R-induced decrease in Bcl-2 levels, as previously reported. *Sirt3* knockout, however, abolished the ability of PCB2 to rescue Bcl-2 expression after I/R injury (Fig. 4f–h). Additionally, we used an siRNA targeting *SIRT3* to knock down SIRT3 expression in A549 cells. As expected, the results of the FACS apoptosis analysis, TUNEL staining and living cell caspase-3 activity assay were to those observed in vivo (Fig. 4i–m). Taken together, SIRT3 is not only a protective factor against lung I/R injury but also an indispensable mediator of the protective effects of PCB2.

**Fig. 5:**
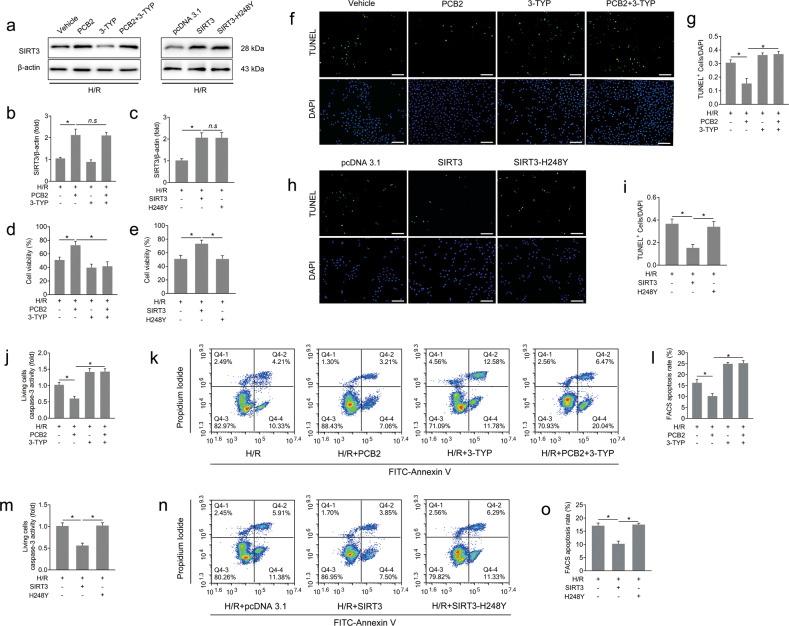
PCB2-mediated pulmonary protection depends on the enzymatic activity of SIRT3. A549 cells were pretreated with 3-TYP (50 μM, 12 h) and/or PCB2 (20 μM, 24 h) in the corresponding groups before H/R treatment. Cells in the pcDNA3.1, SIRT3 and SIRT3-H248Y groups were transfected with empty plasmid, SIRT3 expression plasmid or SIRT3-H248Y plasmid, respectively, and then subjected to H/R injury. All groups of cells were subjected to 6 h of hypoxia followed by 2 h of reoxygenation to induce H/R injury. **a**–**c** SIRT3 protein levels, *n* = 3. **d**, **e** Cell viability, *n* = 6. **f** and **h** Representative images of TUNEL staining in A549 cells; the nuclei were stained with DAPI. Scale bar = 200 μm. **g** and **i** TUNEL scores. The relative number of apoptotic cells is presented as TUNEL-positive cells/DAPI (*n* = 6). **j** and **m** Caspase-3 activity in living A549 cells. *n* = 6. **k** and **n** Representative FACS dot plots of annexin V and propidium iodide dual labeling. **l** and **o** FACS analysis of the apoptotic rate in A549 cells. The sum of the annexin V-positive population and propidium iodide-positive population is shown. *n* = 3. *p < 0.05, *n.s* indicates no significance. Error bars depict the standard deviations.

We pretreated A549 cells with 3-TYP (a highly selective inhibitor of SIRT3 deacetylase activity) or transfected them with SIRT3 overexpression plasmids with (pcDNA3.1-SIRT3, SIRT3) or without deacetylase activity (pcDNA3.1-SIRT3-H248Y, H248Y) to verify whether the pulmonary protection of PCB2 depends on the enzymatic activity of SIRT3 (Fig. 5a). PCB2 + 3-TYP or H248Y transfection did not alter SIRT3 expression compared to the PCB2 group or the SIRT3 overexpression group (Fig. 5b, c). Consistent with the results described above, PCB2 increased the viability of cells upon H/R insult. However, this effect was significantly abrogated in the SIRT3 inactivation group following 3-TYP administration (Fig. 5d). Furthermore, 3-TYP weakened the decrease in the proportion of TUNEL-positive cells, the reduction in the apoptosis rate detected using FACS and the suppression of the caspase-3 activity in living cells induced by PCB2 (Fig. 5f, g, j–l). In the cells transfected with the SIRT3 expression vector, assays for cell viability, TUNEL staining, FACS apoptosis assay and caspase-3 activity revealed protective effects similar to those induced by PCB2. However, we did not observe this level of effective protection against H/R injury in the cells transfected with SIRT3-H248Y, a catalytically inactive SIRT3 mutant (Fig. 5e, h-i, m–o). Thus, we concluded that the deacetylase activity of SIRT3 may play a key role in PCB2-induced antiapoptotic effect on lung I/R injury.

### SIRT3 deacetylates PKM2 at the K433 site in the mitochondria

**Fig. 6:**
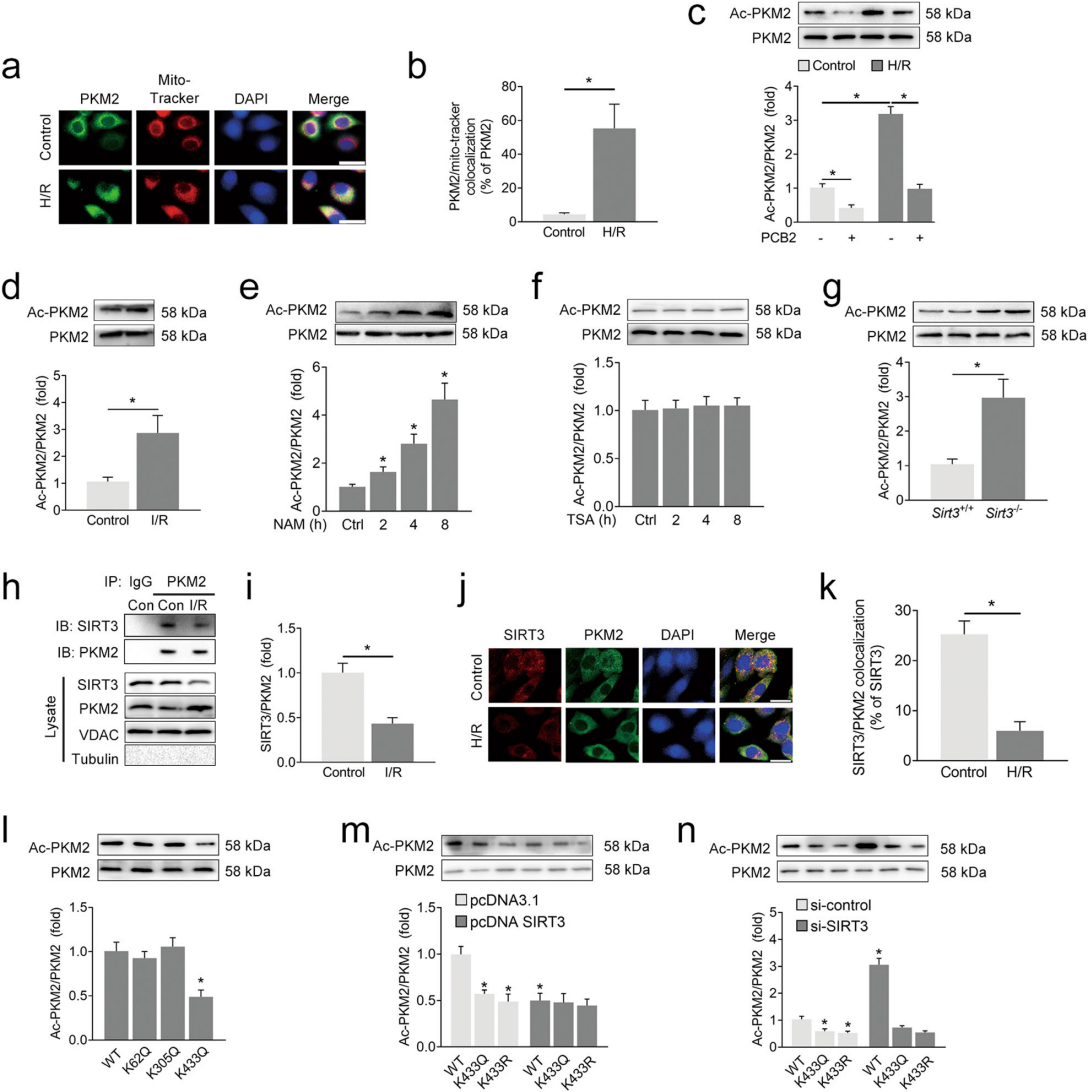
SIRT3 deacetylates PKM2 at the K433 site in the mitochondria. **a**, **b** A549 cells were subjected to 6 h of hypoxia followed by 2 h of reoxygenation to induce H/R injury. Immunofluorescence analyses were performed using anti-PKM2 antibodies or MitoTracker probes. Representative images are shown in (**a**). Scale bar = 25 μm; colocalization data are shown in (**b**). **c**, **d** A549 cells were pretreated with PCB2 (20 μM) for 24 h and subjected to 6 h of hypoxia followed by 2 h of reoxygenation (H/R); C57BL/6 mice were subjected to 1 h of ischemia followed by 2 h of reperfusion to establish lung I/R injury. Level of acetylated PKM2. *n* = 3. **e**, **f** Level of acetylated PKM2 in A549 cells after treatment with NAM (10 mM) or TSA (0.5 μM) for 2 to 8 h, *n* = 3. **g** Level of acetylated PKM2 in the lungs from the *Sirt3* wild-type (*Sirt3*^+/+^) or *Sirt3* knockout (*Sirt3*^-/-^) mice, *n* = 3. **h**, **i** Representative coimmunoprecipitation blots showing the interaction of SIRT3 with PKM2 in the lungs from the control (Con) or I/R mice. The lysate represents the mitochondrial protein extracts used for immunoprecipitation. IB, immunoblotting; IP, immunoprecipitation; IgG, negative control. *n* = 3. **j**, **k** A549 cells were subjected to 6 h of hypoxia followed by 2 h of reoxygenation to induce H/R injury. Immunofluorescence analyses were performed using anti-SIRT3 or anti-PKM2 antibodies. Representative images are shown in (**j**). Scale bar = 25 μm; colocalization data are shown in (**k**). **l** PKM2 acetylation in A549 cells transfected with acetylation site-specific mutants of PKM2, *n* = 3. **m** A549 cells were cotransfected with acetylation site-specific mutants of PKM2 and a SIRT3 expression plasmid (pcDNA SIRT3) or negative control (pcDNA3.1). Levels of acetylated PKM2. *n* = 3. **n** A549 cells were cotransfected with acetylation site-specific mutants of PKM2 and an siRNA targeting *SIRT3* (si-SIRT3) or negative control (si-control). Levels of acetylated PKM2. *n* = 3. *p < 0.05 compared with the first group or the indicated group. Error bars depict the standard deviations.

SIRT3 is the primary deacetylase targeting mitochondrial substrates to protect cells from various stresses, including apoptosis [41]. In the mitochondria, PKM2 is recognized as an important antiapoptotic regulator, and PKM2 deficiency results in Bcl-2 degradation and apoptotic cell death. As shown in Fig. 6a, b, increased levels of PKM2 accumulated in the mitochondria upon H/R treatment, as confirmed by immunofluorescence staining with an anti-PKM2 antibody and the MitoTracker probe. Because PKM2 is deacetylated by members of the sirtuin family, resulting in altered enzymatic activity, we conducted additional experiments to determine whether PKM2 is associated with SIRT3 deacetylation-induced protection against lung I/R injury in vitro and in vivo. In the mitochondrial lysate, an acetyl-lysine immunoprecipitation assay showed that the levels of acetylated PKM2 were significantly increased after H/R treatment in vitro (Fig. 6c), and this increase was identical to that observed in vivo (Fig. 6d). In addition, the sirtuin inhibitor NAM increased the level of acetylated PKM2, whereas TSA, a histone deacetylase inhibitor, did not produce a similar increase (Fig. 6e, f). Based on these findings, mitochondrial PKM2 is hyperacetylated after H/R injury and that this process may depend on NAD^+^-dependent mechanisms.

SIRT3 is the major NAD^+^-dependent deacetylase in the mitochondria that may be regulated by PCB2. In the groups treated with PCB2, the levels of acetylated PKM2 were substantially reduced in both the control and H/R-treated cells (Fig. 6c). Thus, we further examined the potential interaction between SIRT3 and PKM2. Interestingly, the level of acetylated mitochondrial PKM2 in the *Sirt3*-knockout mice was significantly increased compared with that in their wild-type counterparts (Fig. 6g). In *Sirt3* wild-type mice, PCB2 relieved SIRT3 suppression after I/R injury while simultaneously decreasing the level of acetylated PKM2. However, we did not observe a similar result in *Sirt3*-knockout mice, and the ability of PCB2 to inhibit PKM2 acetylation was substantially reduced in the absence of SIRT3 expression (Supplementary Fig. 4). Immunoblotting analyses of SIRT3 immunoprecipitated with an anti-PKM2 antibody showed that mitochondrial PKM2 clearly interacted with SIRT3 in the control mice, while this interaction was significantly decreased after I/R injury (Fig. 6h, i). These observations were further supported by immunofluorescence staining, showing that PKM2 exhibited stronger colocalization with SIRT3 in the control cells than in the H/R-treated cells (Fig. 6j, k). Thus, SIRT3 may interact with and deacetylate mitochondrial PKM2 during lung I/R injury.

Previous reports identified three acetylation sites of PKM2: K62, K305 and K433. K62 is not conserved, while K305 and K433 are conserved among mammals [17, 42]. We generated individual mutants of both sites and transfected A549 cells with these mutants. As shown in Fig. 6l, only transfection of the K433Q mutant reduced the acetylation of PKM2 in A549 cells. In the cells cotransfected with wild-type PKM2 (WT) and SIRT3 overexpression or knockdown constructs, the level of acetylated PKM2 was decreased or increased accordingly, respectively; however, neither SIRT3 overexpression (Fig. 6m) nor SIRT3 knockdown (Fig. 6n) altered the level of acetylated PKM2 in the A549 cells transfected with K433Q or K433R. These results suggested that the K433 site might be the main deacetylation target of SIRT3 on PKM2 in A549 cells.

### The protective effect of SIRT3 on lung I/R injury partially depends on PKM2

**Fig. 7:**
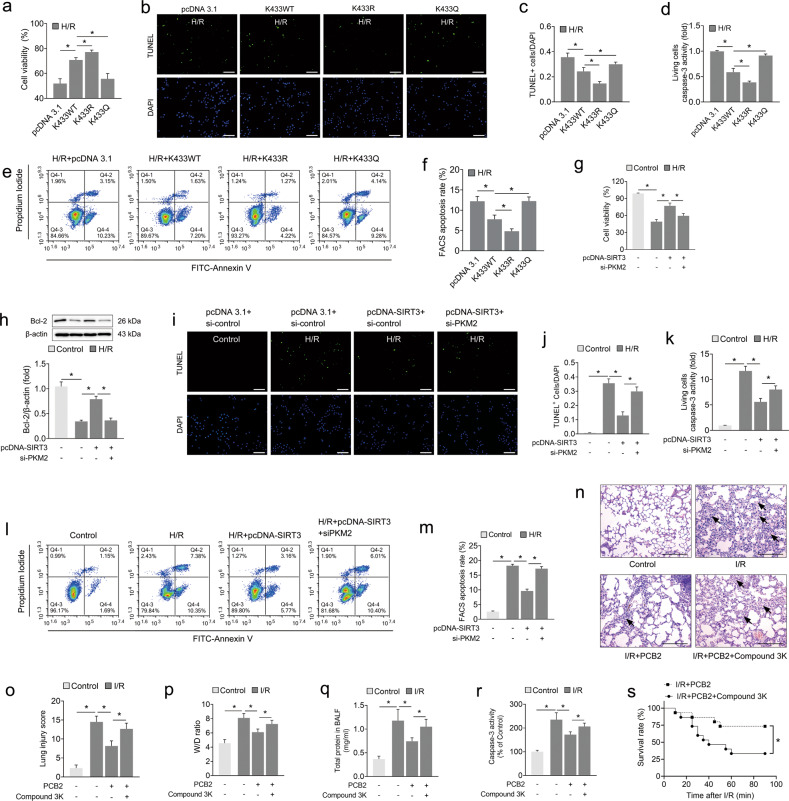
The protective effect of SIRT3 on lung I/R injury partially depends on PKM2. **a**–**f** A549 cells were cotransfected with acetylation site-specific mutants of PKM2 or the negative control (pcDNA3.1) and then subjected to 6 h of hypoxia followed by 2 h of reoxygenation to induce H/R injury. **a** Cell viability, *n* = 6. **b** Representative images of TUNEL staining in A549 cells; the nuclei were stained with DAPI. Scale bar = 200 μm. **c** The relative number of apoptotic cells is presented as TUNEL-positive cells/DAPI (*n* = 6). **d** Caspase-3 activity in living A549 cells. *n* = 6. **e**, **f** FACS analysis of the apoptotic rate in A549 cells. The sum of the annexin V-positive population and propidium iodide-positive population is shown. *n* = 3. **g**–**m** A549 cells were cotransfected with the SIRT3 expression plasmid (pcDNA SIRT3), siRNA targeting *PKM2* (si-PKM2) or the corresponding negative control. Then, the cells were subjected to 6 h of hypoxia followed by 2 h of reoxygenation to achieve H/R injury. **g** Cell viability, *n* = 6. **h** Bcl-2 protein level, *n* = 3. **i** Representative images of TUNEL staining in A549 cells; the nuclei were stained with DAPI. Scale bar = 200 μm. **j** The relative number of apoptotic cells is presented as TUNEL-positive cells/DAPI (*n* = 6). **k** Caspase-3 activity in living A549 cells. *n* = 6. **l**, **m** FACS analysis of the apoptotic rate in A549 cells. The sum of the annexin V-positive population and propidium iodide-positive population is shown. *n* = 3. **n**–**s** C57BL/6 mice were subjected to 1 h of ischemia followed by 2 h of reperfusion to establish lung I/R injury; mice from the control group underwent sham surgical procedures. PCB2 (30 mg/kg) and/or compound 3 K (10 mg/kg) were injected intraperitoneally once a day for three consecutive days before the operation. **n** Representative images of H&E-stained lung tissues. Scale bar = 200 μm. Black arrows indicate representative pathological damage. **o** Histopathological scores (Mikawa’s score) of the lung, *n* = 6. **p**–**r** The W/D ratio, total protein concentration in BALF and caspase-3 activity in the lung, *n* = 6. **s** The survival rate of the I/R mice, *n* = 15. *p < 0.05. Error bars depict the standard deviations.

Next, we aimed to validate whether PKM2 and its acetylation status are involved in SIRT3-mediated protection against lung I/R injury. The transfection of wild-type PKM2 (K433WT) and PKM2 with mutation of the K433 lysine residue to arginine (deacetylation mimic of PKM2, K433R) clearly increased cell viability after the H/R insult. However, PKM2 with a mutation of lysine to glutamine (acetylation mimic of PKM2, K433Q) failed to exert a protective effect similar to that of K433WT (Fig. 7a). In addition, the K433R mutant more noticeably reduced the proportion of TUNEL-positive cells, attenuated caspase-3 activity in living cells, and decreased the apoptosis rate compared with K433WT, whereas the K433Q mutant did not exert the same protective effect (Fig. 7b–f).

We confirmed whether PKM2 is a downstream mediator of pulmonary epithelial H/R injury through SIRT3 deacetylation by cotransfecting A549 cells with a SIRT3 overexpression plasmid (pcDNA-SIRT3) and siRNA targeting *PKM2* (si-PKM2) prior to the H/R insult. As expected, SIRT3 overexpression increased cell viability and Bcl-2 protein expression after H/R injury (Fig. 7g, h). In addition, SIRT3 overexpression also reduced the proportion of TUNEL-positive nuclei and caspase-3 activity in living cells and decreased the apoptosis rate detected using FACS (Fig. 7i–m). Interestingly, these protective effects afforded by SIRT3 overexpression were substantially abolished when PKM2 was simultaneously knocked down (Fig. 7g–m). Collectively, since SIRT3 deacetylates PKM2 at K433 and this deacetylation exerts a protective effect on H/R injury in A549 cells, we further speculate that the protective effects of SIRT3 may be partially mediated by SIRT3-dependent PKM2 deacetylation.

We coadministered a specific inhibitor of PKM2, compound 3 K, and PCB2, and established a lung I/R injury model to further verify the aforementioned protective role of PKM2 associated with the PCB2-mediated activation of the SIRT3 pathway in vivo. Interestingly, PKM2 inhibition decreased the ability of PCB2 to ameliorate histological lesions, the changes in the W/D ratio, increased BALF protein levels and caspase-3 activation in the I/R-injured lung (Fig. 7n–r). Furthermore, the PCB2 and compound 3 K coadministration group exhibited a lower survival rate than the mice treated with PCB2 alone after lung I/R injury. Thus, we concluded that PCB2 protects the lung from I/R injury, especially apoptotic injury, at least partially through the SIRT3-PKM2 deacetylation pathway (Fig. 7n).

## Discussion

Lung I/R injury is a life-threatening clinical syndrome that often occurs after cardiopulmonary surgery, lung transplantation and pulmonary embolism [1, 3, 43]. Extensive evidence, including biopsies from human transplanted lungs, confirmed that apoptosis is an important cell death pathway that is activated after reperfusion, which contributes to the high complication and mortality rates [1, 6, 7]. In the present study, we first reported the findings described below. 1) The SIRT3/PKM2 pathway is a protective target for the suppression of apoptosis during the pathogenesis of lung I/R injury. 2) The K433 residue might be the dominant acetylation site on PKM2 that is involved in SIRT3-induced antiapoptotic activity, and the protection induced by SIRT3 partially depends on the deacetylation of mitochondrial PKM2. 3) Modulation of the SIRT3/PKM2 pathway by PCB2 may result in significant protective effects on lung I/R injury.

Apoptosis has been shown to play an important role in the pathogenesis of I/R injury in a variety of organs, such as the brain, heart and lung [6, 7, 44, 45]. Unlike necrosis, apoptosis is a tightly regulated cell death program that involves the caspase cascade and the Bcl-2 family [46, 47]. Many researchers have focused on delineating and regulating apoptotic mechanisms during lung I/R injury and aimed to develop efficient treatments for patients suffering from complications after lung transplantation and pulmonary embolism. In this regard, previous reports have confirmed that apoptosis is initiated shortly after the onset of ischemia and then continues during the early stage of reperfusion [6, 7]. The results from the present study are consistent with those from previous studies, showing that noticeable apoptosis was observed in the reperfused lung and H/R-treated cells. Interestingly, PCB2 substantially attenuated apoptosis by suppressing caspase-3 activation, inducing Bcl-2 expression, and reducing the TUNEL-positive rate and apoptosis rate observed using FACS. These effects eventually led to significant increases in pulmonary oxygenation and animal survival. Therefore, we suggest that PCB2 is a potent regulator of apoptosis that protects against lung I/R injury. Some researchers hypothesized that apoptosis should also be viewed as an adaptive mechanism to eliminate injured cells and avoid more destructive necrosis during reperfusion. However, up to 34% of pulmonary cells undergo apoptotic death during reperfusion after human lung transplantation [6], and excessive cell death will inevitably lead to pulmonary dysfunction and an increase in mortality. Thus, early and effective interventions targeting apoptosis, such as the PCB2 pretreatment in this study, may be an effective prophylactic approach to treat lung I/R injury.

Abnormalities in the expression and posttranslational modification of mitochondrial proteins affect energy production, impair redox homeostasis and induce apoptotic cell death [20, 23, 48]. Among the mammalian sirtuin family members expressed in the mitochondria (SIRT3, SIRT4, and SIRT5), SIRT3 is the major regulator of the mitochondrial acetylome and targets more than 65% of mitochondrial proteins [41]. In addition to the traditional role of SIRT3 as a redox sensor, accumulating evidence has linked SIRT3 to I/R injury, with a critical role in competing with apoptosis [23, 24]. *Sirt3*^-/-^ mice suffered more serious apoptotic injury and worse organ dysfunction after myocardial and hepatic I/R than control mice [24, 49]. In our recent study, *Sirt3* deficiency weakened reactive oxygen species (ROS) scavenging and aggravated epithelial apoptosis during murine intestinal I/R injury [23]. Here, we revealed that pulmonary SIRT3 expression is significantly downregulated by I/R injury. As expected, *Sirt3* knockout in mice further exacerbated I/R-induced histopathological damage, pulmonary dysfunction and apoptotic injury and increased the animal death rate. In the in vitro model, more obvious TUNEL-positive staining, an increase in the apoptosis rate detected using FACS and caspase-3 activation were observed in *SIRT3* knockdown A549 cells under H/R conditions. Thus, we concluded that SIRT3 plays a key role in regulating pulmonary I/R-mediated apoptosis.

Although the antioxidant and free radical scavenging properties of polyphenols are well established, they have more recently been shown to exert strong modulatory effects on the sirtuin family [50]. We detected the ability of several polyphenols to regulate SIRT3 expression induced by lung I/R injury in our experimental mice (Supplementary Fig. 1a; detailed chemical structures are shown in Fig. 1b). PCB2 ameliorated I/R-induced SIRT3 suppression in the lung and upregulated SIRT3 expression in A549 cells in both a dose- and time-dependent manner. Although PCB2 administration relieved H/R injury similar to SIRT3 overexpression, it did not show obvious protection in the absence of SIRT3 in vivo and in vitro. These data indicated that PCB2 may be a potent activator of SIRT3, and the protective effect of PCB2 is at least partially mediated by self-induced SIRT3 activation. Notably, protocatechuic acid (PCA), another polyphenol compound, also showed a strong ability to upregulate hepatic SIRT3 in our recent study [31]. However, PCA was not better than PCB2 at promoting SIRT3 expression in the lung (data not shown). The detailed mechanisms require further investigation in the future.

Neither SIRT3 enzymatic inhibition (3-TYP) nor transfection of an inactivated SIRT3 expression plasmid (SIRT3-H248Y) achieved effective protection against H/R-induced apoptosis, although these procedures did not impair the expression of the SIRT3 protein. These results indicated that the deacetylase activity of SIRT3 may play an indispensable role in protecting against lung I/R injury. In this study, we first investigated the function of SIRT3-dependent deacetylation and activation of PKM2, an antiapoptotic factor expressed in mitochondria. Under oxidative stress, PKM2 translocates to mitochondria and enhances resistance to apoptosis by stabilizing Bcl-2 [16]. Here, we found that PKM2 indeed accumulated in the mitochondria upon H/R treatment. Interestingly, the inhibitory effects of NAM, I/R and *Sirt3* knockout on SIRT3 expression increased the level of acetylated PKM2 in the mitochondria. After verification using coimmunoprecipitation (co-IP) and immunofluorescence staining, we concluded that SIRT3 directly binds and deacetylates PKM2 in the mitochondria. By screening mutants of the common acetylation sites on PKM2, we identified K433 as the main site of SIRT3-mediated deacetylation. Functionally, the simulation of acetylation by PKM2-K433Q substantially diminished the antiapoptotic effect of PKM2 overexpression on H/R-injured A549 cells, while PKM2-K433R magnified the corresponding protective effect. Thus, the K433 residue might be the dominant acetylation site on PKM2 that is involved in SIRT3-mediated protection against lung I/R injury. Furthermore, PKM2 knockdown abolished the antiapoptotic and Bcl-2 rescue effects induced by SIRT3 overexpression. In vivo, compound 3 K, a specific inhibitor of PKM2, also decreased PCB2-induced pulmonary protection. Thus, the deacetylation of PKM2 by SIRT3 may be the key mechanism that helps to prevent lung I/R-induced apoptosis. These findings, combined with those from a previous study [16], showed that the antiapoptotic activity of PKM2 is not only induced by mitochondrial translocation and stabilizing Bcl-2 but also depends on deacetylation of the K433 site by SIRT3.

This study still has some limitations. 1) The pulmonary epithelium plays a critical role in the lung by facilitating gas exchange and providing defense against airborne pathogens. Researchers should target epithelial cells for therapeutic interventions to prevent I/R injury. Of course, other cell types, such as macrophages, natural killer cells, and endothelial cells, are also known to mediate lung I/R injury. More sophisticated in vitro models containing multiple cell types should be developed in future studies. 2) In addition to acetylation, previous reports have documented that PKM2 contains phosphorylation and ubiquitination sites. SIRT3 crosstalk with the acetylation/phosphorylation/ubiquitination of PKM2 or other mechanisms may affect its enzymatic activity, and further investigations are necessary to investigate this issue.

**Fig. 8:**
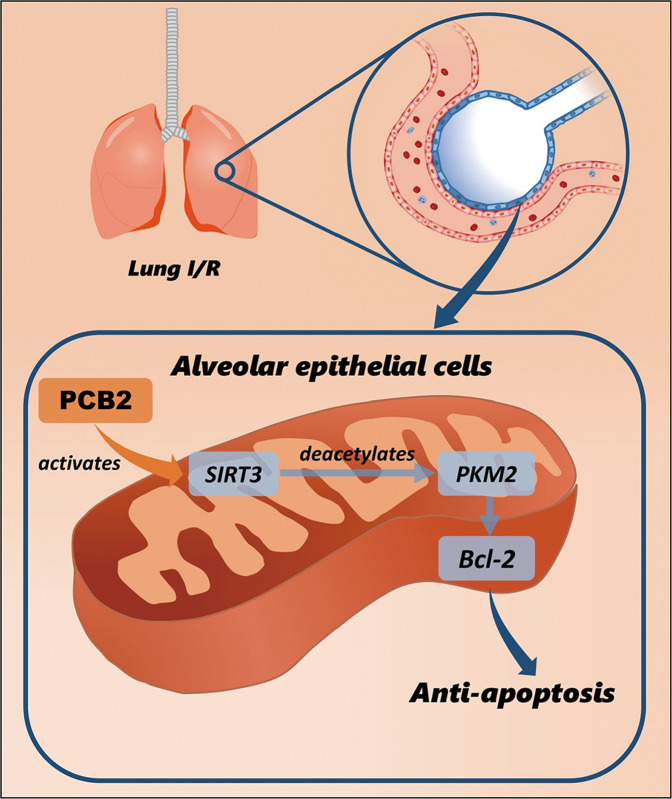
Modulation of the SIRT3/PKM2 pathway by PCB2 may result in an antiapoptotic effect on lung I/R injury. Schematic representation of PCB2-mediated activation of the SIRT3/PKM2 deacetylation pathway to prevent apoptosis in the lung after I/R injury.

In summary, our study provides the first evidence for the important role of the SIRT3/PKM2 pathway in lung I/R-induced apoptosis (a schematic is shown in Fig. 8). The activation of SIRT3 by PCB2 alleviates the pulmonary dysfunction induced by I/R involving the deacetylation of PKM2. Furthermore, SIRT3-mediated deacetylation of PKM2 at residue K433 decreases apoptosis in a lung I/R injury model. Thus, SIRT3/PKM2 is a critical signaling pathway involved in lung I/R injury, and our results provide key mechanistic insights into lung I/R injury.

## Supplementary information

Supplementary Materials

Original Data File

Checklist

## Data Availability

The datasets generated during the study are available from the corresponding author upon reasonable request.
