# Supplementary material for: Mitochondrial PKM2 deacetylation by procyanidin B2-induced SIRT3 upregulation alleviates lung ischemia/reperfusion injury
Source: Cell Death Dis. 2022 Jul 11;13(7):594. doi: 10.1038/s41419-022-05051-w (PMC9276754; doi:10.1038/s41419-022-05051-w)
Supplement: Supplementary file 1 — Supplementary Materials [file 41419_2022_5051_MOESM1_ESM.pdf]

Supplementary Figures

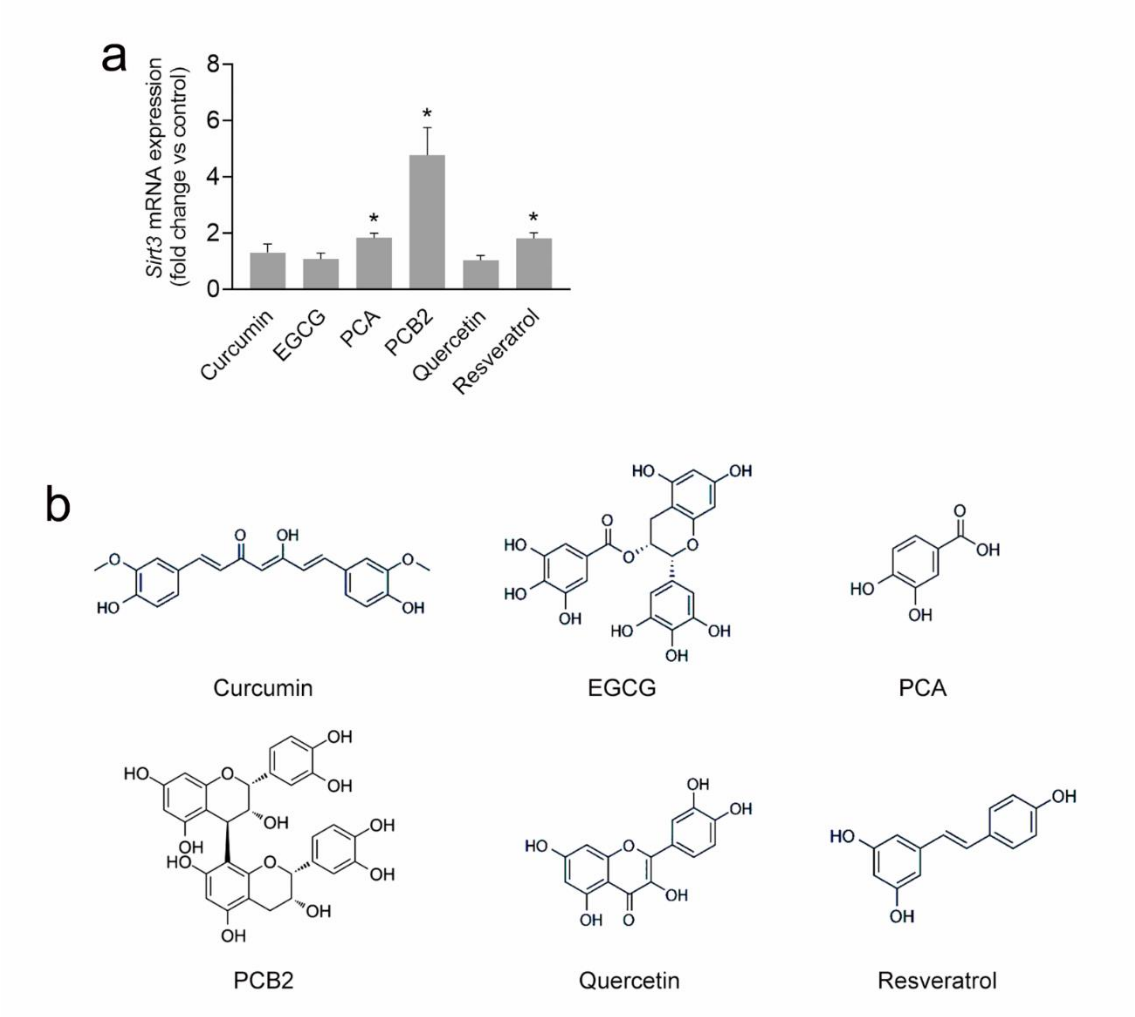

Supplementary Fig. 1 PCB2 administration upregulates *Sirt3* mRNA expression.

PCB2 (30 mg/kg), curcumin (25 mg/kg), (-)-epigallocatechin gallate (EGCG, 85 mg/kg), protocatechuic acid (PCA, 30 mg/kg), quercetin (50 mg/kg), resveratrol (10 mg/kg) or vehicle was injected intraperitoneally into mice once a day for three consecutive days. (a) The fold change in *Sirt3* mRNA expression in the lung. n=3. *p<0.05 compared with each of the vehicle controls. (b) Chemical structures of curcumin, EGCG, PCA, PCB2, quercetin and resveratrol.

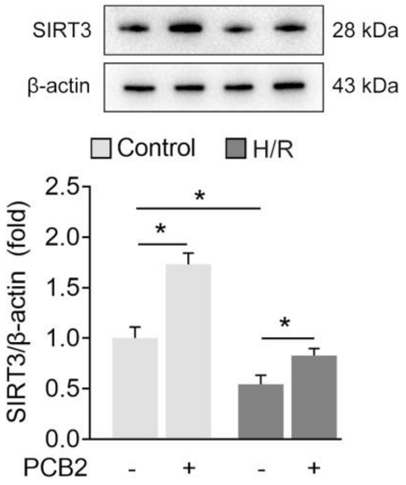

Supplementary Fig. 2 PCB2 relieves H/R-induced SIRT3 suppression in A549 cells.

A549 cells were treated with 20 μM PCB2 for 24 h and then subjected to 6 h of hypoxia followed by 2 h of reoxygenation to induce H/R injury. The SIRT3 protein level in A549 cells is shown, *n*=3. **p*<0.05. Error bars depict the standard deviations.

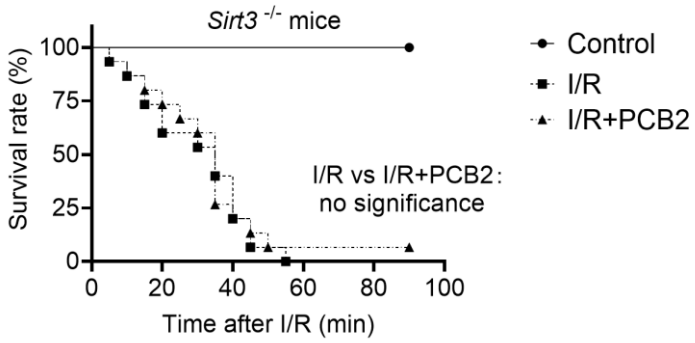

Supplementary Fig. 3 PCB2 administration did not improve the survival rate of *Sirt3* knockout mice after lung I/R injury.

Sirt3 knockout mice were subjected to 1 h of ischemia followed by 2 h of reperfusion to induce lung I/R injury. Mice from the control group underwent sham surgical procedures. PCB2 (30 mg/kg) was injected intraperitoneally for three consecutive days before the operation. The survival rate of the mice is shown, *n*=15.

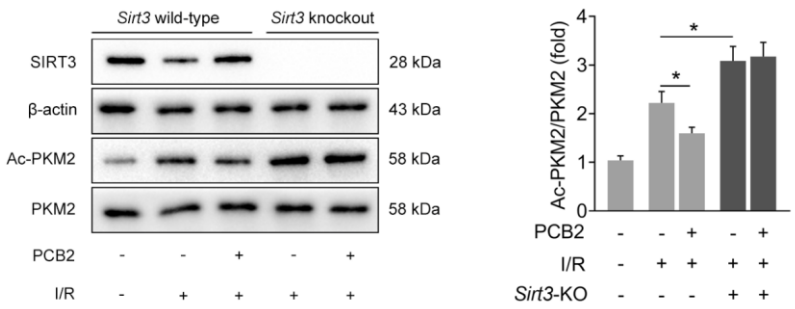

Supplementary Fig. 4 PCB2-induced PKM2 deacetylation depends on SIRT3 activation.

Mice were subjected to 1 h of ischemia followed by 2 h of reperfusion to induce lung I/R injury. Mice from the control group underwent sham surgical procedures. PCB2 (30 mg/kg) was injected intraperitoneally for three consecutive days before the operation. The level of the SIRT3 protein and the level of acetylated PKM2 in the lungs from *Sirt3* wild-type or *Sirt3* knockout mice are shown, *n*=3. Error bars depict the standard deviations.

Supplementary Table 1 Primer sequences

Gene	Forward primer (5'-3')	Reverse primer (5'-3')
Sirt1	GCTGACGACTTCGACGACG	TCGGTCAACAGGAGGTTGTCT
Sirt2	GCCTGGGTTCCCAAAAGGAG	GAGCGGAAGTCAGGGATACC
Sirt3	ATCCCGGACTTCAGATCCCC	CAACATGAAAAAGGGCTTGGG
Sirt4	GGCGACGTGTTCCTCACTG	ACAAAGTCAACCTTGTCTGGG
Sirt5	CTCCGGGCCGATTCATTTCC	GCGTTCGCAAAACACTTCCG
Sirt6	ATGTCGGTGAATTATGCAGCA	GCTGGAGGACTGCCACATTA
Sirt7	AGCATCACCCGTTTGCATGA	GGCAGTACGCTCAGTCACAT
β-actin	AGAGGGAAATCGTGCGTGAC	AGAGGGAAATCGTGCGTGAC
