# Supplementary material for: Mitochondrial PKM2 deacetylation by procyanidin B2-induced SIRT3 upregulation alleviates lung ischemia/reperfusion injury
Source: Cell Death Dis. 2022 Jul 11;13(7):594. doi: 10.1038/s41419-022-05051-w (PMC9276754; doi:10.1038/s41419-022-05051-w)

Fig. 2

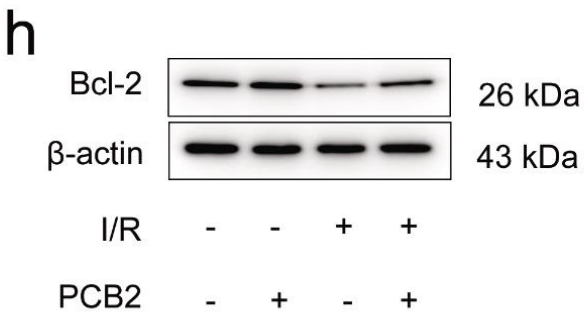

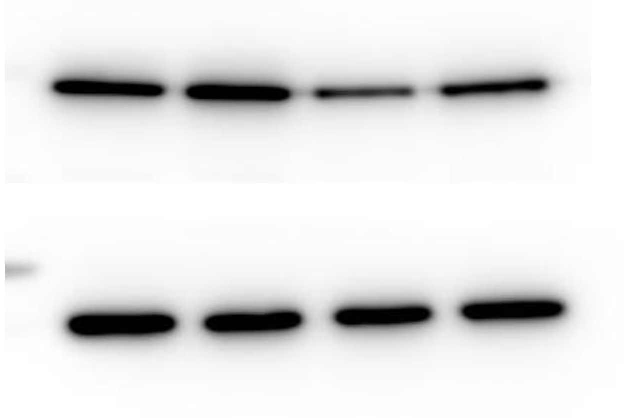

Fig. 3

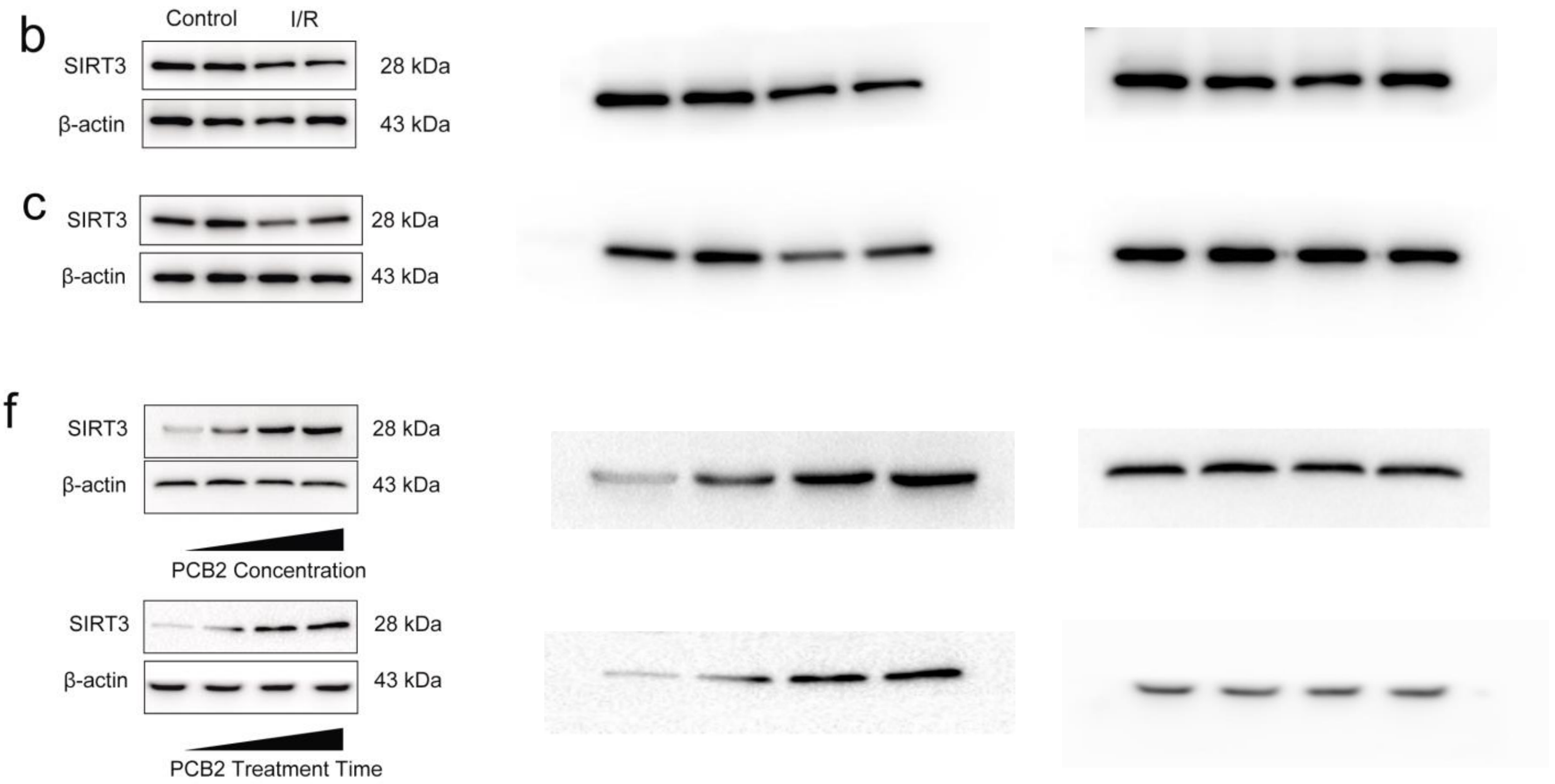
b
Control
I/R
SIRT3
28 kDa
β-actin
43 kDa
c
SIRT3
28 kDa
β-actin
43 kDa
f
SIRT3
28 kDa
β-actin
43 kDa
PCB2 Concentration
SIRT3
28 kDa
β-actin
43 kDa
PCB2 Treatment Time

Fig. 4

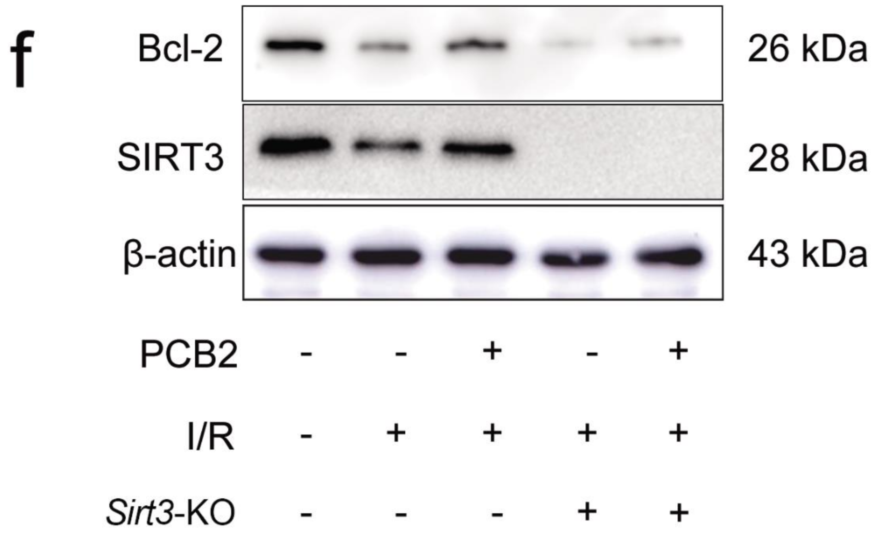

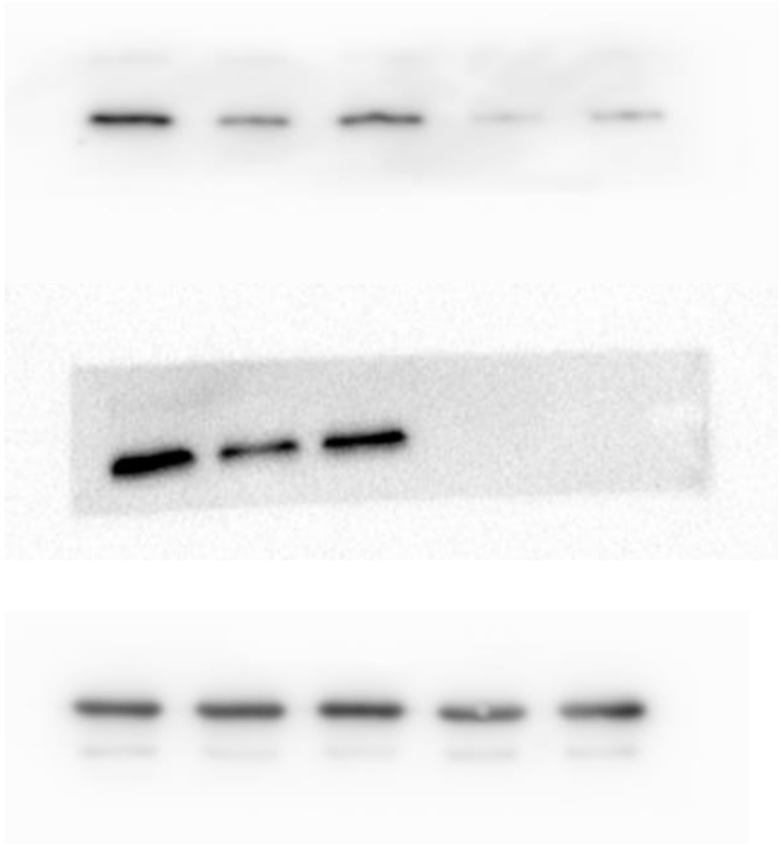

Fig. 5

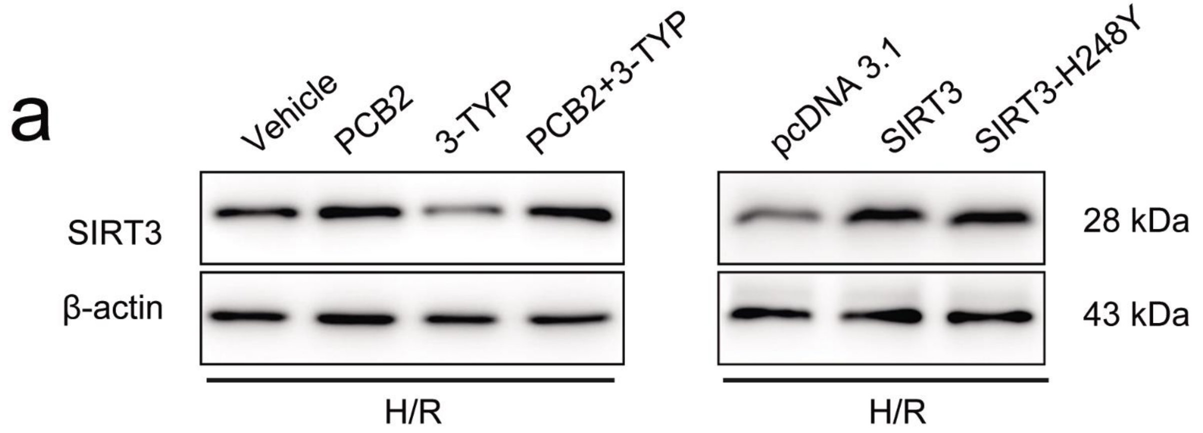
a
Vehicle
PCB2
3-TYP
PCB2+3-TYP
pcDNA 3.1
SIRT3
SIRT3-H248Y
SIRT3
β-actin
28 kDa
43 kDa
H/R
H/R

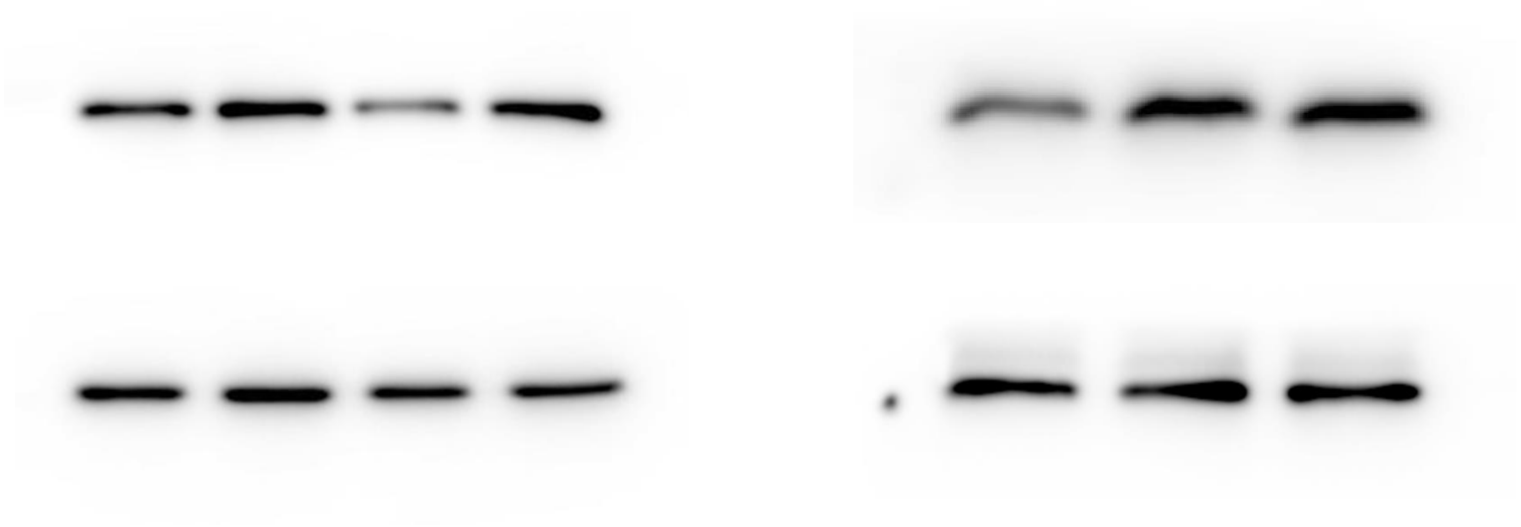

Fig. 6

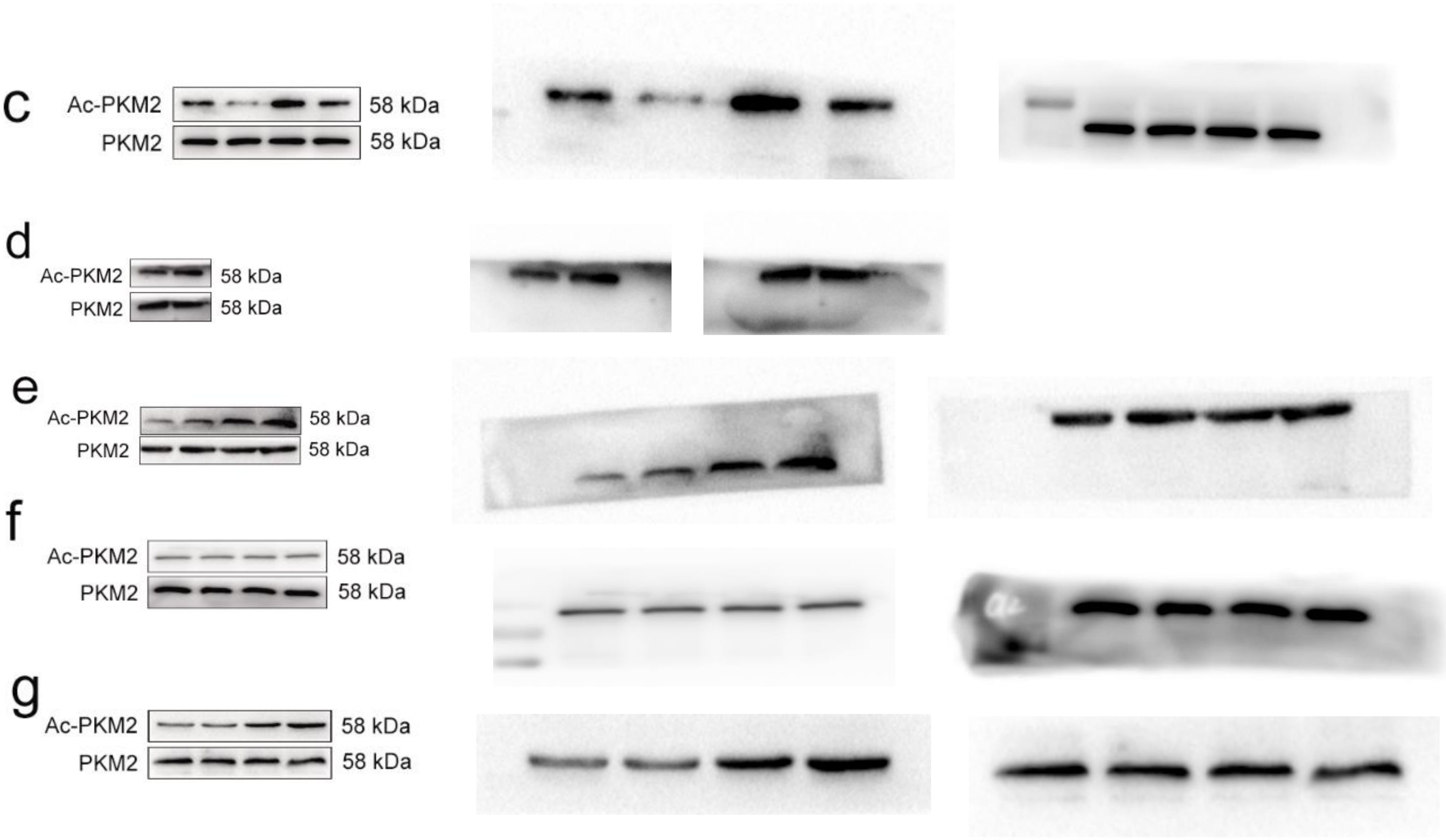
c
Ac-PKM2
58 kDa
PKM2
58 kDa
d
Ac-PKM2
58 kDa
PKM2
58 kDa
e
Ac-PKM2
58 kDa
PKM2
58 kDa
f
Ac-PKM2
58 kDa
PKM2
58 kDa
g
Ac-PKM2
58 kDa
PKM2
58 kDa

Fig. 6

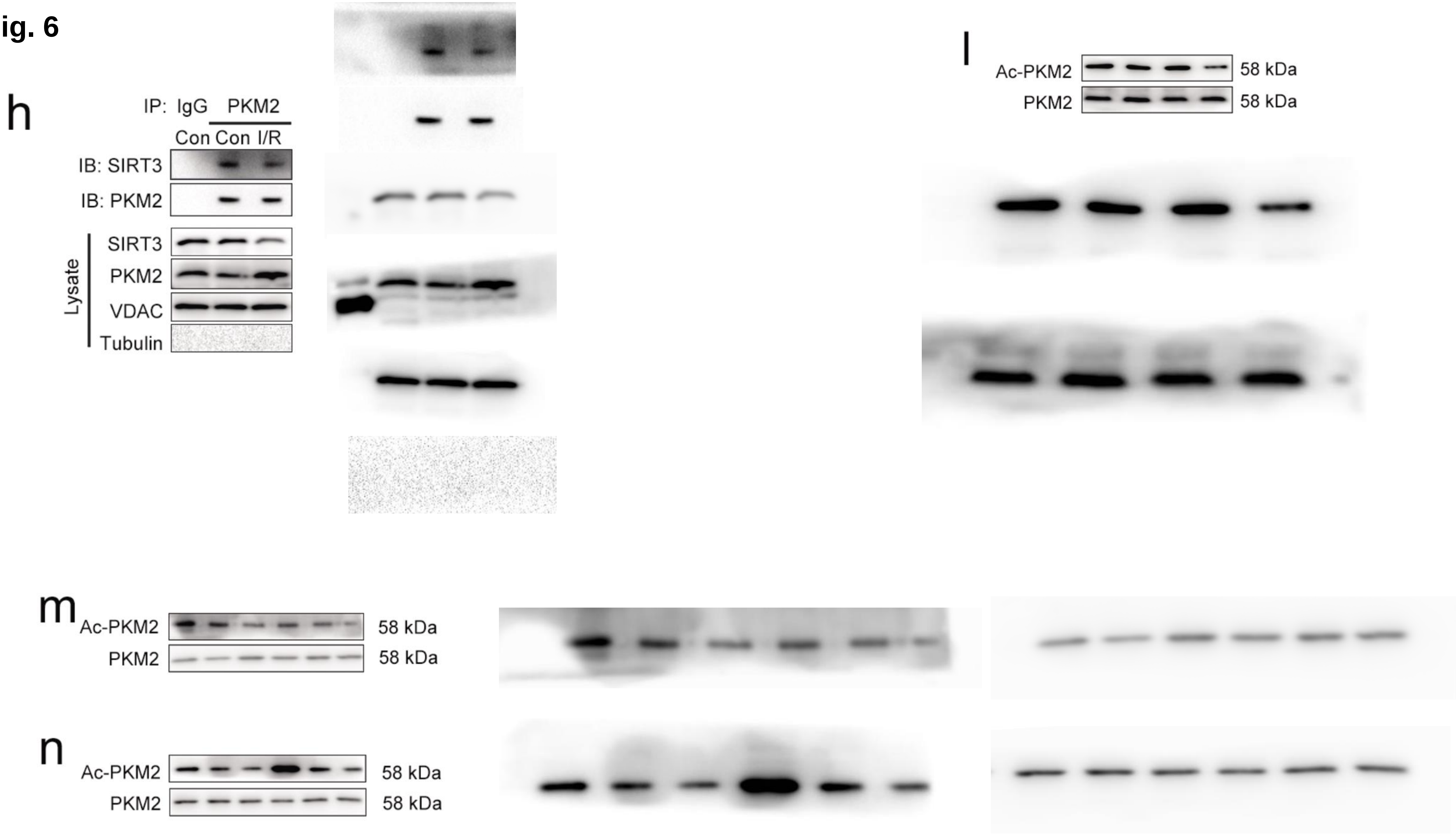
h
IP: IgG PKM2
Con Con I/R
IB: SIRT3
IB: PKM2
Lysate
SIRT3
PKM2
VDAC
Tubulin
l
Ac-PKM2 58 kDa
PKM2 58 kDa
m
Ac-PKM2 58 kDa
PKM2 58 kDa
n
Ac-PKM2 58 kDa
PKM2 58 kDa

Fig. 7

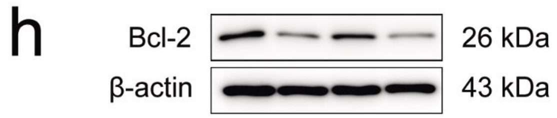

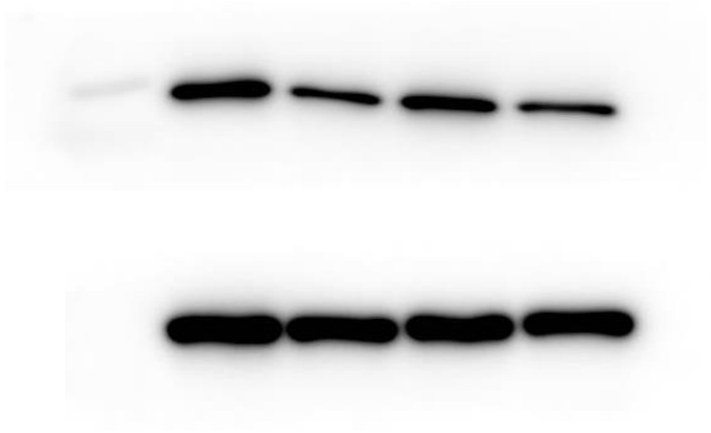

Supplementary Fig. 2

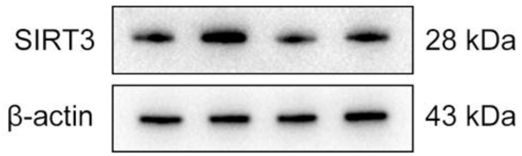

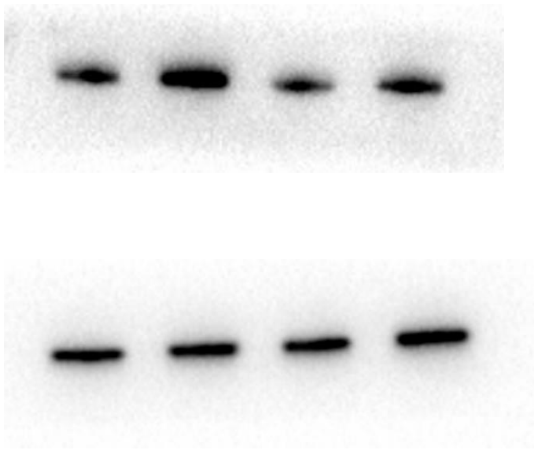

Supplementary Fig. 4

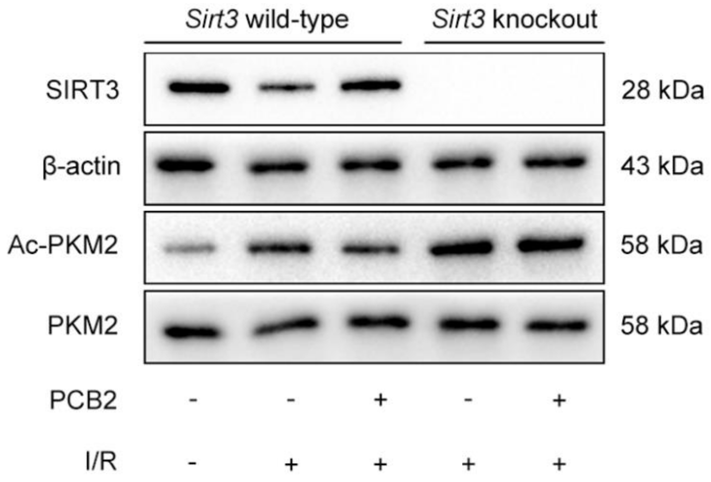

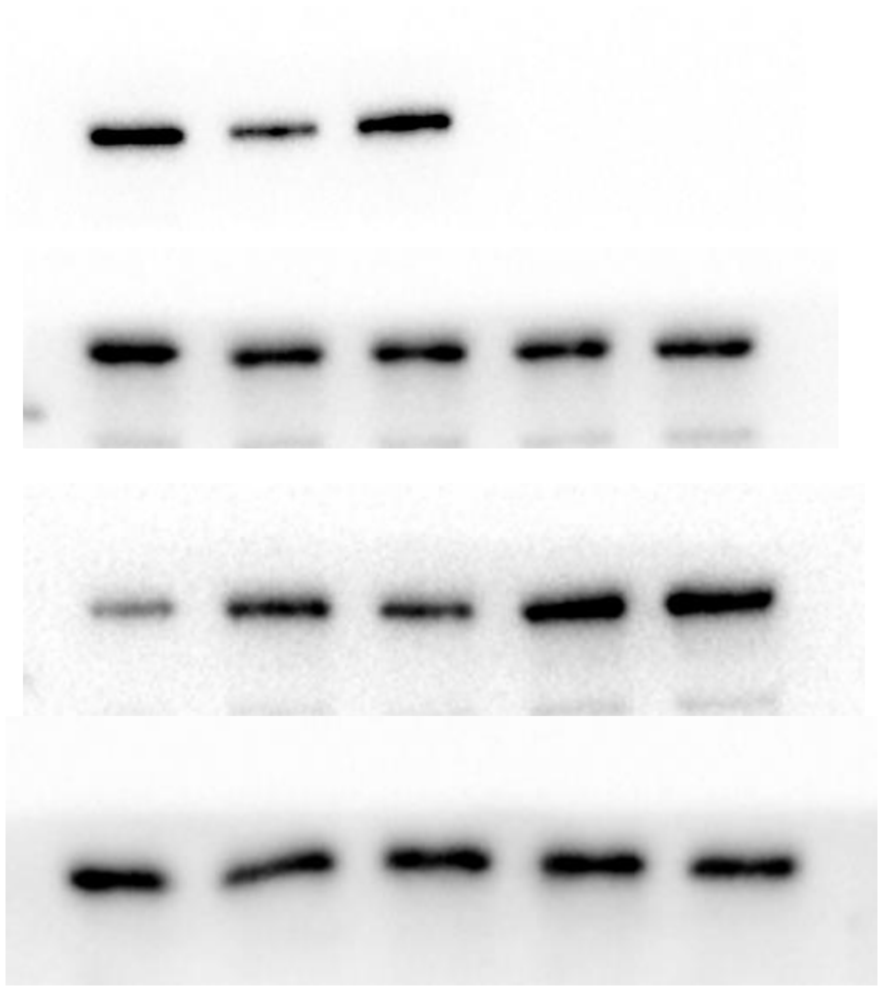

Supplement: Supplementary file 2 — Original Data File [file 41419_2022_5051_MOESM2_ESM.pdf]
